# Adaptive optics in single objective inclined light sheet microscopy enables three-dimensional localization microscopy in adult *Drosophila* brains

**DOI:** 10.3389/fnins.2022.954949

**Published:** 2022-10-06

**Authors:** Shih-Te Hung, Arnau Llobet Rosell, Daphne Jurriens, Marijn Siemons, Oleg Soloviev, Lukas C. Kapitein, Kristin Grußmayer, Lukas J. Neukomm, Michel Verhaegen, Carlas Smith

**Affiliations:** ^1^Delft Center for Systems and Control, Delft University of Technology, Delft, Netherlands; ^2^Department of Fundamental Neurosciences, University of Lausanne, Lausanne, Switzerland; ^3^Cell Biology, Neurobiology and Biophysics, Department of Biology, Faculty of Science, Utrecht University, Utrecht, Netherlands; ^4^Department of Bionanoscience, Kavli Institute of Nanoscience Delft, Delft University of Technology, Delft, Netherlands; ^5^Department of Imaging Physics, Delft University of Technology, Delft, Netherlands

**Keywords:** Super-resolution Microscopy, localization microscopy, adaptive optics, *Drosophila*, brain

## Abstract

Single-molecule localization microscopy (SMLM) enables the high-resolution visualization of organelle structures and the precise localization of individual proteins. However, the expected resolution is not achieved in tissue as the imaging conditions deteriorate. Sample-induced aberrations distort the point spread function (PSF), and high background fluorescence decreases the localization precision. Here, we synergistically combine sensorless adaptive optics (AO), *in-situ* 3D-PSF calibration, and a single-objective lens inclined light sheet microscope (SOLEIL), termed (AO-SOLEIL), to mitigate deep tissue-induced deteriorations. We apply AO-SOLEIL on several dSTORM samples including brains of adult *Drosophila*. We observed a 2x improvement in the estimated axial localization precision with respect to widefield without aberration correction while we used synergistic solution. AO-SOLEIL enhances the overall imaging resolution and further facilitates the visualization of sub-cellular structures in tissue.

## 1. Introduction

Single-molecule localization microscopy (SMLM) routinely surpasses the diffraction limit in thin samples (Lidke et al., 2005; Betzig et al., 2006; Hess et al., 2006; Egner et al., 2007; Manley et al., 2008). This is achieved by estimating the position of the isolated fluorescence molecules with higher precision than the diffraction limit (Smith and Joseph, 2010). For example, SMLM revealed the periodic structure of actin filaments in axons (Xu et al., 2012), and the distribution of βII spectrin in dendrites (Zhong et al., 2014) in cultured neurons. While these approaches uncover substantial cell-intrinsic details, the effects of extracellular components, such as the extracellular matrix (ECM), cannot be studied *in-vitro* (Kapałczyńska et al., 2016). The absence of the ECM can affect cellular morphology and gene/protein expression (Sanyal, 2017), revealing the limitations of imaging cultured cells. However, the application of SMLM inside *ex-vivo* tissue is also limited due to sample-induced aberrations and high fluorescent background. The refractive index mismatch between the immersion media and tissue induces (high-order) spherical aberrations (Booth and Wilson, 2001). This is particularly problematic for SMLM due to the use of a high NA oil immersion objective lens to optimize the emission fluorescence efficiency. Sample-induced aberrations distort the point spread function (PSF), which can result in artifact-containing reconstructions. Furthermore, imaging in tissue often increases background fluorescence, which results in a decrease in the localization precision (Smith and Joseph, 2010), and thus a decrease in the theoretical maximum spot detection efficiency (Smith et al., 2015). A decrease in the localization precision and the localization density ultimately results in a lower reconstruction resolution (Nieuwenhuizen et al., 2013). Thus, increased background fluorescence leads to a lower reconstruction resolution.

To decrease the background fluorescence, several optical sectioning methods were established, including highly inclined and laminated optical sheet (HILO) microscopy (Tokunaga et al., 2008), and variable-angle epi-fluorescence (VAEM) microscopy (Konopka and Bednarek, 2008) that adopt an inclined widefield illumination profile to achieve optical sectioning at a sub-10μm level. However, the size of the field of view (FOV) is around tens of μm in HILO microscopy, which limits the application of HILO microscopy. Alternatively, selective plane illumination microscopy (SPIM) is widely used to achieve optical sectioning. SPIM relies on two orthogonal objectives, where one illuminates the sample while the other one collects the fluorescence (Ahrens et al., 2013; Lu et al., 2019). Tilted light-sheet microscopy (TILT3D) successfully utilizes a high detection NA objective by illuminating the sample with a tilted light sheet (Gustavsson et al., 2018) in combination with PSF engineering. However, the sample mounting is challenging with dual objective lens configurations because of the need for customized sample holders. Oblique plane microscopy (OPM) alleviates this drawback by the use of the objective for illumination and detection. Optical sectioning is achieved by illuminating the sample with an inclined light sheet. However, OPM requires multiple objective lenses downstream in the emission path to rotate the focal plane matching the inclined light-sheet illumination (Dunsby, 2008; Kumar and Kozorovitskiy, 2019, 2020; Yang et al., 2019; An et al., 2020; Sapoznik et al., 2020). Single objective lens inclined light sheet (SOLEIL) microscopy is based on an oblique light-sheet with optimal optical sectioning (Hung et al., 2022). The focal plane is not re-positioned by additional objectives; a deformable mirror (DM) is used for PSF engineering instead. SOLEIL is therefore also compatible with 3D SMLM.

To avoid artifacts in SMLM reconstructions an accurate point spread function model (PSF) is needed (Babcock and Zhuang, 2017; Aristov et al., 2018; Li et al., 2018). To mitigate the effects from sample-induced aberrations both numerical (McGorty et al., 2014) and pre-calibration (Tafteh et al., 2015; Cabriel et al., 2018; Li et al., 2019) approaches have been used for modeling depth-dependent PSFs. Both approaches neglect sample-induced aberrations originating from biological variability. To accommodate for this higher order sample-induced aberrations, the PSF should be retrieved from *in-situ* data. The state-of-the-art for *in-situ* PSF calibration is INSPR (Xu et al., 2020).

Sample-induced aberrations deteriorate the localization precision (Mlodzianoski et al., 2018). Therefore, in 3D SMLM, *in-situ* PSF calibration should be combined with active aberration correction. To accomplish sample-induced aberration correction, two distinct approaches have been adopted: the first is based on a wave-front sensor to measure sample-induced aberrations and a DM to compensate for the measured aberration (Park et al., 2021); the second on a sensorless approach (Burke et al., 2015; Tehrani et al., 2015; Mlodzianoski et al., 2018; Siemons et al., 2021), where the aberrations are minimized by maximizing a metric for the image quality. Both algorithm and metric function should be tailored to an application to avoid non-convergent aberration corrections (Siemons et al., 2021) due to the non-convex dependency of the aberrations (Debarre et al., 2007; Soloviev, 2020).

**Figure 1:**
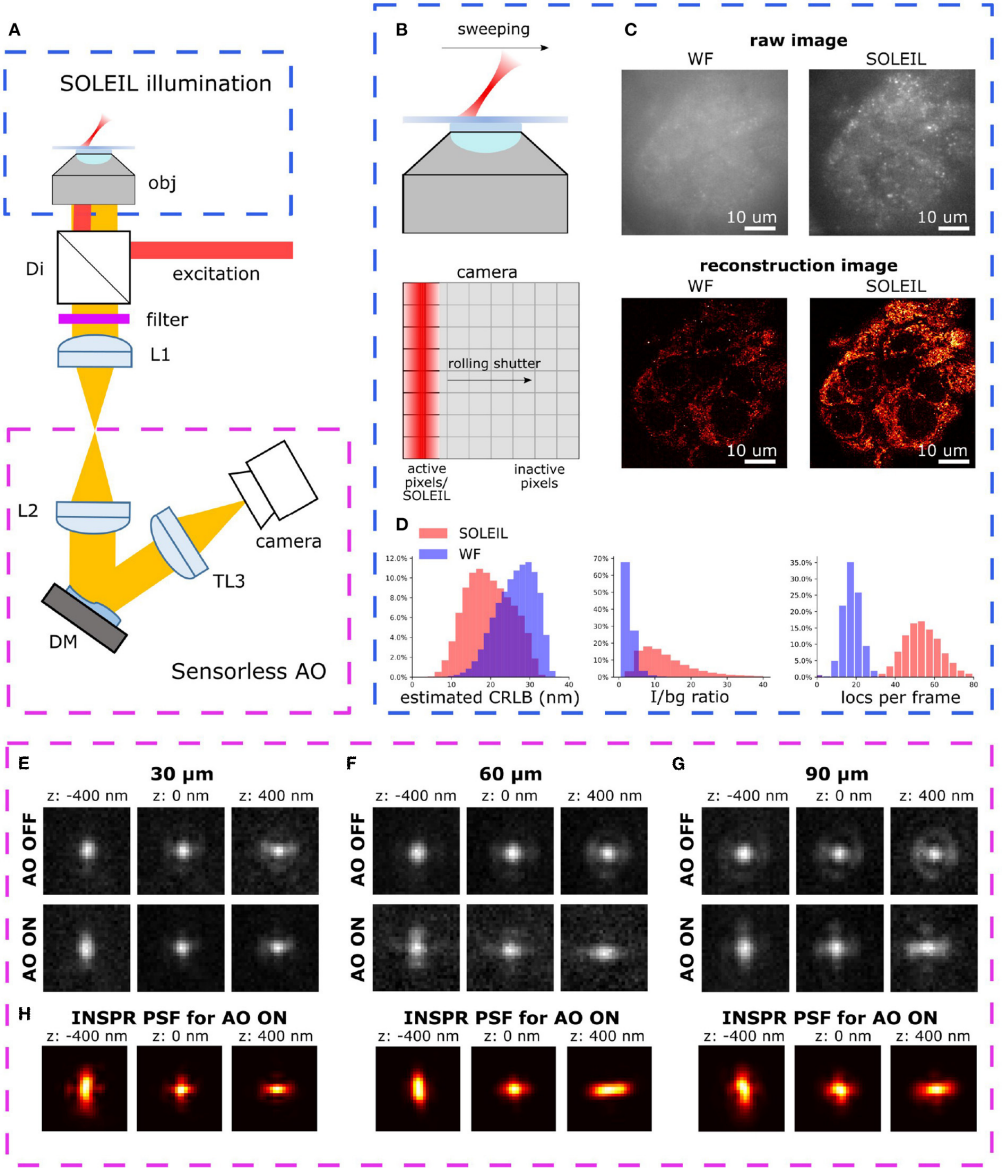
**(A)** Optical setup of AO-SOLEIL microscopy. obj: objective lens, Di: dichroic mirror, filter: emission filter, L1 to L3: achromatic doublet lens, DM: deformable mirror, camera: sCMOS camera. For the details of system, see Supplementary Section 1. **(B)** Working principle of SOLEIL and camera readout. The camera readout is synchronized with the SOLEIL illumination area (red). The SOLEIL illumination is controlled by a galvo mirror. **(C)** Demonstration of SOLEIL background reduction (dSTORM) on spheroid Caco2-BBE cells (mitochondria). upper row: raw camera image by widefield and SOLEIL microscopy (equal contrast). lower row: SMLM reconstructions. **(D)** Comparison between WF and SOLEIL of estimated lateral CRLB, I/bg ratio, and number of localization per frame (loc per frame). **(E–G)** The astigmatism PSF before (AO OFF) and after (AO ON) AO correction at different imaging depth (30, 60, and 90 μm). The PSFs were acquired by imaging 23 nm fluorescence beads (Thermo Fisher Scientific, Tetraspeck) embedded in 1% agarose gel. The emission wavelength is 680 nm. **(H)** The estimated *in-situ* PSF models using the INSPR algorithm.

To alleviate the difficulties of 3D SMLM in tissue we propose AO-SOLEIL, which combines adaptive optics, *in-situ* PSF calibration, and three-dimensional SOLEIL microscopy (Figure 1). We experimentally show the need for AO-SOLEIL as sample-induced aberrations deteriorate the axial localization precision and thereby prevent three-dimensional localization microscopy. To correct for sample-induced aberrations and enable 3D SMLM in tissue, we implemented sensorless adaptive optics combined with *in-situ* PSF calibration. We demonstrate the feasibility of AO-SOLEIL with several samples, including mitochondria in Caco2-BBE cells, and single neurons in the adult *Drosophila* brain. The results show that AO-SOLEIL facilitates the visualization of sub-cellular structures in tissue.

## 2. AO-SOLEIL

### 2.1. Principle of AO-SOLEIL

We proposed a synergistical approach (AO-SOLEIL) to optimize for three-dimensional SMLM in tissue. The AO-SOlEIL consists of two modules, SOLEIL illumination and sensorless AO (Figure 1A). AO-SOLEIL adopts single objective lightsheet illumination (SOLEIL) for background rejection Hung et al. (2022). Here we synchronized the camera readout pixel and the SOLEIL illumination to expose the whole FOV on a same image frame (Figure 1B). The ability of background rejection is demonstrated by imaging spheroid Caco2-BBE sample, which is a 10 μm thick cell sample (Figures 1C,D). From the raw images, we observed that SOLEIL delivers a higher signal to background ratio (Figure 1C). Furthermore, the reconstruction image from SOLEIL microscopy shows more details than the reconstruction image from widefield microscopy (Figure 1C). We observed that the background rejection delivers a higher detection efficiency and an improved CRLB (Figure 1D). To restore the 3D PSF and improve the CRLB, the 7 Zernike modes ($Z_{2}^{\pm 2}$, $Z_{3}^{\pm 1}$, $Z_{3}^{\pm 3}$, $Z_{4}^{0}$) are corrected using the DM (Figures 1E–G). To enable unbiased estimation the 3D PSF model is estimated directly from the raw SMLM data (Figure 1H). For this *in-situ* PSF calibration we estimated up to the fifth radial order of Zernike modes using the INSPR algorithm (Xu et al., 2020).

We observed that we could restore the astigmatism PSF up to 90 μm depth (Figure 1H).

### 2.2. Sensorless AO algorithm

In this manuscript, we use a model-based wavefront sensorless approach for aberration correction. Namely, the aberration in the pupil is represented as a linear combination of the first 7 Zernike modes ($Z_{2}^{\pm 2}$, $Z_{3}^{\pm 1}$, $Z_{3}^{\pm 3}$, $Z_{4}^{0}$), and the indirect wavefront sensing is based on the widely accepted (e.g., Debarre et al., 2007; Žurauskas et al., 2019) Fourier annulus image quality metric.

The metric, defined by two radii *r*_1_ and *r*_2_, is given by relative total energy of the image spatial frequencies **m** in the annulus with inner and outer radii given by *r*_1_ and *r*_2_. In practice, for a digital image *I*[*x, y*] and its 2D discrete Fourier Transform $\hat{\text{I}}\left[m,n\right]$,


(1) $$\begin{array}{l} \hat{\text{I}}\left[m,n\right]=F_{2}I\left[x,y\right], \end{array}$$

the metric $M\left(\hat{\text{I}}\left[m,n\right]\right)=M\left(\hat{\text{I}}\left[m,n\right];r_{1},r_{2}\right)$ is defined as


(2) $$\begin{array}{l} M\left(\hat{\text{I}}\left[m,n\right]\right)=\frac{\underset{m,n}{\sum }|\hat{\text{I}}\left[m,n\right]|\cdot w\left[m,n\right]}{\underset{m,n}{\sum }|\hat{\text{I}}\left[m,n\right]|\cdot u\left[m,n\right]}, \end{array}$$

with binary masks *w, u* defined as


(3) $$\begin{array}{l} w[m,n]=\{\begin{array}{ll} 1, & r_{1}<\;|m|\;<r_{2}\\ 0, & \text{else} \end{array},\\ u[m,n]=\{\begin{array}{ll} 1, & |m|\;<R\\ 0, & \text{ else} \end{array}, \end{array}$$

where $|\text{m}|=\sqrt{m^{2}+n^{2}},$ and *R* is the radius corresponding to the diffraction-limited maximum spatial frequency. (For a camera with pixel size Δ*x* and resolution *L* × *L*, $R=\frac{\text{NA}}{\lambda }L\Delta x$.)

The idea is to discard the low spatial frequencies (background variations) and the high spatial frequencies (usually attributed to noise). Radii *r*_1_, *r*_2_ are often expressed in multiples of *R* (Mlodzianoski et al., 2018; Siemons et al., 2021).

In our work, to achieve sensorless AO correction of spherical aberration, we have used the previous published metric function with *r*_1_ = 0, *r*_2_ = *R*/2 (Mlodzianoski et al., 2018).

As the image *I* depends on the aberration, represented by its Zernike coefficients vector $\vec{\alpha }$, the metric $M\left(\hat{\text{I}}\left[m,n\right]\right)$ is also a function of α, $M=M\left(\vec{\alpha }\right)$, As the higher values of the metric correspond to a sharper image (Žurauskas et al., 2019), the aberration correction is equivalent to the optimisation problem


(4) $$\begin{array}{l} \hat{\vec{\alpha }}=\underset{\vec{\alpha }}{\text{arg max }}M\left(\vec{\alpha }\right). \end{array}$$

The optimisation is performed independently for each of the Zernike modes used to control the DM. To this end, for each of the modes we acquired 11 images with the mode amplitude values varying uniformly in the range [−λ/2, λ/2] and computing the image metric for each of the images. Previously it has been shown that a minimum of 2*N*+1 of measurements are needed for correcting *N* Zernike modes with sensorless AO correction (Debarre et al., 2007; Žurauskas et al., 2019). With more advanced algorithm, N+1 measurements are also possible to achieve AO correction (Antonello et al., 2012; Booth, 2006). In our experiments a minimum of 11 measurement steps are needed to mitigate a high fluorescence background and sparse images (**Figure 9**).

The correction point for each mode was determined by fitting the metric value points with a Gaussian function *G*(M) and taking its central point. Namely, with *G*(M) defined as


(5) $$\begin{array}{l} G\left(\text{M}\right)=ae^{\frac{-{\left(\text{M}-c\right)}^{2}}{\sigma^{2}}}+bg, \end{array}$$

where M is the metric value, *a* is the amplitude of Gaussian function, σ is the width of Gaussian function, *c* is the center of Gaussian function, which is the value for aberration correction, and *bg* is the background of the metric value curve. The optimal value ${\hat{\alpha }}_{i}$ for the current mode is *c*.

We used bounded non-linear least squares to fit the amplitude term *a*, center *c*, width σ, and background *bg*. Based on the design of the metric function, we know the best aberration correction happens at the peak of the metric value curve, so the amplitude term must be positive. The boundary condition of amplitude term is 0 < *a* < ∞. The fitting procedure is done by using the *curve fit* function in the Scipy library (version 3.8). We corrected the spherical aberration first and then we corrected the other first order aberrations. In our samples we mainly observed spherical aberration because of refractive index mismatch. Therefore, first two iterations were performed to correct for the spherical aberration ($Z_{4}^{0}$). Subsequently, to correct for the other aberrations ($Z_{2}^{\pm 2}$, $Z_{3}^{\pm 1}$, $Z_{3}^{\pm 3}$) one iteration were done for each mode. In total, the aberration correction uses 88 frames, which is a small fraction in typical dSTORM imaging (10,000 ~ 30,000 frames).

### 2.3. Defocus compensation for spherical aberration correction

**Figure 2:**
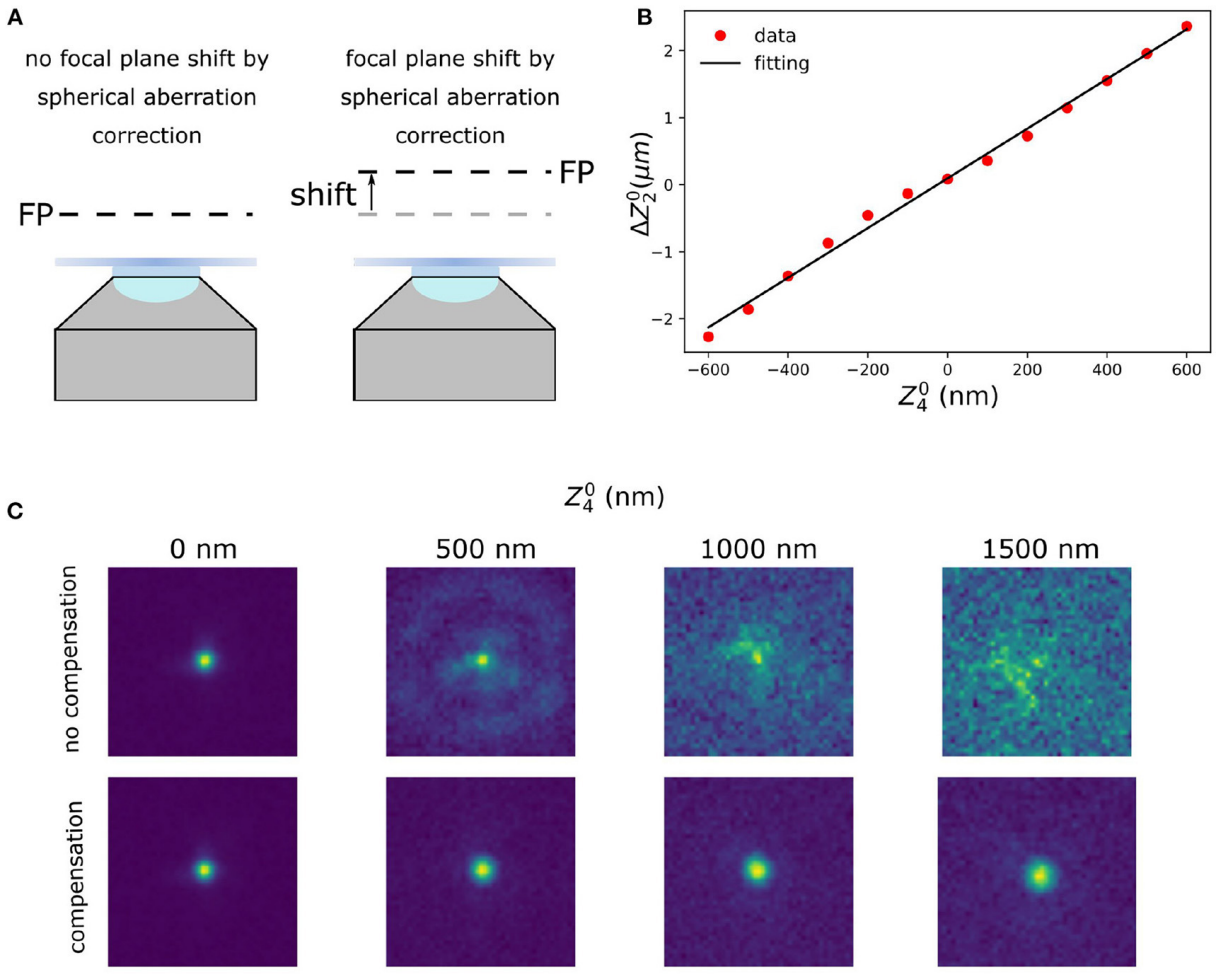
Defocus calibration for spherical aberration compensation. **(A)** Focal plane shift induced by coupling between defocus and spherical aberration. When correcting for the spherical aberration, the coupling between the spherical and the defocus aberration shifts the focal plane (FP). **(B)** Calibration of the compensation for the defocus introduced by spherical aberration. **(C)** Raw data with different amount of spherical aberration (${\text{Z}}_{4}^{0}$: 0, 500, 1,000, and 1,500 nm) without (upper row) and with (lower row) defocus compensation. The calibration was done with spherical aberration in the range of –600 ~ 600 nm.

Zernike polynomials, although orthogonal in the phase of the pupil plane, do not guarantee the absence of cross-talk in the optimisation procedure described in the previous subsection. That means that by maximising $M\left(\vec{\alpha }\right)$ moving along one of the modes, we might move out of the maximum value for the other modes. Different techniques are known to deal with this effect, finding their principles in Gram-Schmidt orthogonalization (Debarre et al., 2007; Soloviev, 2020). In this work, we propose the following simple procedure to establish and to compensate for the major cross-talk effect in our setup, that is between the defocus and spherical aberration terms (Figure 2).

To compensate for the axial defocus that is caused by a DM when correcting for spherical aberrations a calibration is made (Mlodzianoski et al., 2018). To create this calibration the defocus offset was measured by imaging fluorescent beads (23 nm; embedded in 1% agarose) on a 2D surface, which means the fluorescence beads only appear at a certain axial plane. This was done as follows: a certain amount of spherical aberration is introduced on the DM. This shifted the focal plane and we then applied a defocus aberration to re-focus the bead images. To determine the amount of defocus for the re-focusing we used the same metric function as for sensorless AO. This procedure was repeated for a different amounts of spherical aberration. Finally, a linear function was fitted by minimizing the mean square error (Figure 2B), which was used as the calibration:


(6) $$\begin{array}{l} \Delta {\text{Z}}_{2}^{0}=\beta \cdot {\text{Z}}_{4}^{0}, \end{array}$$

where ${\text{Z}}_{2}^{0},{\text{Z}}_{4}^{0}$ are defocus and spherical Zernike mode, respectively, $\Delta {\text{Z}}_{2}^{0}$ is the defocus aberration, and β is the *coupling* coefficient, which in our system is 0.00371 μm/nm.

### 2.4. Impact of AO correction on INSPR PSF model and axial localization precision

**Figure 3:**
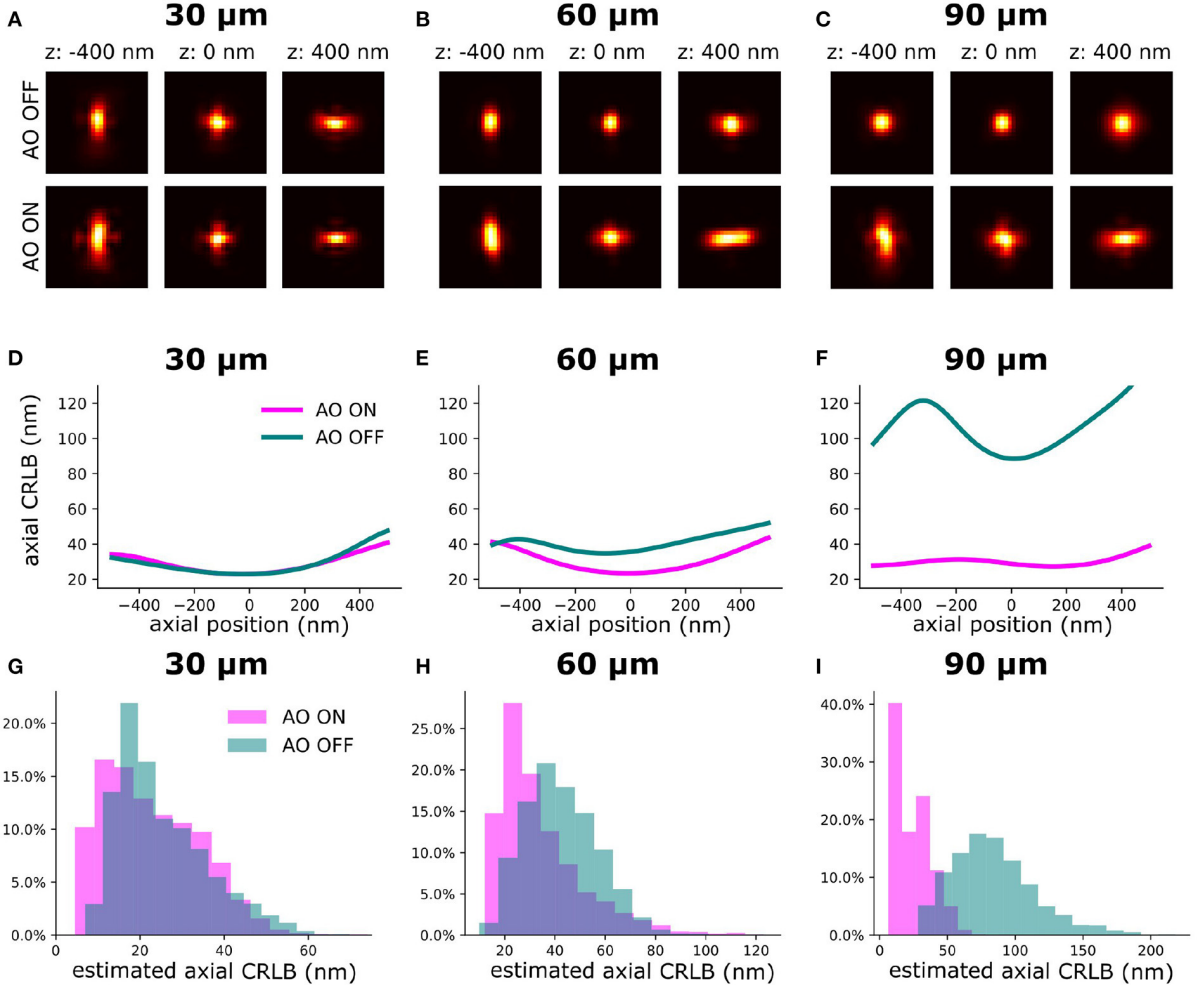
The INSPR PSF before and after sensorless AO correction at different imaging depths. The PSF model was calibrated by imaging a fluorescence bead sample. **(A–C)** Estimated *in-situ* PSF models using the INSPR algorithm with and without AO correction at different depths (30, 60, and 90 μm). **(D–F)** The theoretical axial CRLB based on the estimated PSF model from **(A–C)** with 3,000 photons of intensity and 50 photons/pixel of background. **(G–I)** The distribution of estimated axial CRLB at different imaging depths.

To validate the synergy between our sensorless AO algorithm and the INSPR algorithm for improving the axial CRLB, we imaged homogenously embedded beads (23 nm) in 1% agarose (>100 μm). Firstly, we performed AO correction to minimize the sample-induced aberration. We call this procedure AO ON and without this procedure AO OFF. Then, we introduced an astigmatism aberration with the DM to generate an astigmatism-based PSF for 3D localization. To estimate the *in-situ* PSF, INSPR algorithm needs multiple single-molecules at different axial positions to build a model (Figures 3A–C) (Xu et al., 2020). To estimate the *in-situ* PSF model with the INSPR algorithm we acquired data by moving the piezo stage in discrete steps (100 nm) along an axial and lateral dimension (2 μm). In Figures 3D–F, we show the theoretical axial CRLB and in Figures 3G–J we show the axial CRLB estimated from the localizations. We found that this synergetic combination of AO correction and INSPR can improve the axial CRLB. However, this effect is not observed in lateral CRLB (Supplementary Figure S2).

### 2.5. Validation of sensorless AO in combination with *in-situ* PSF calibration

**Figure 4:**
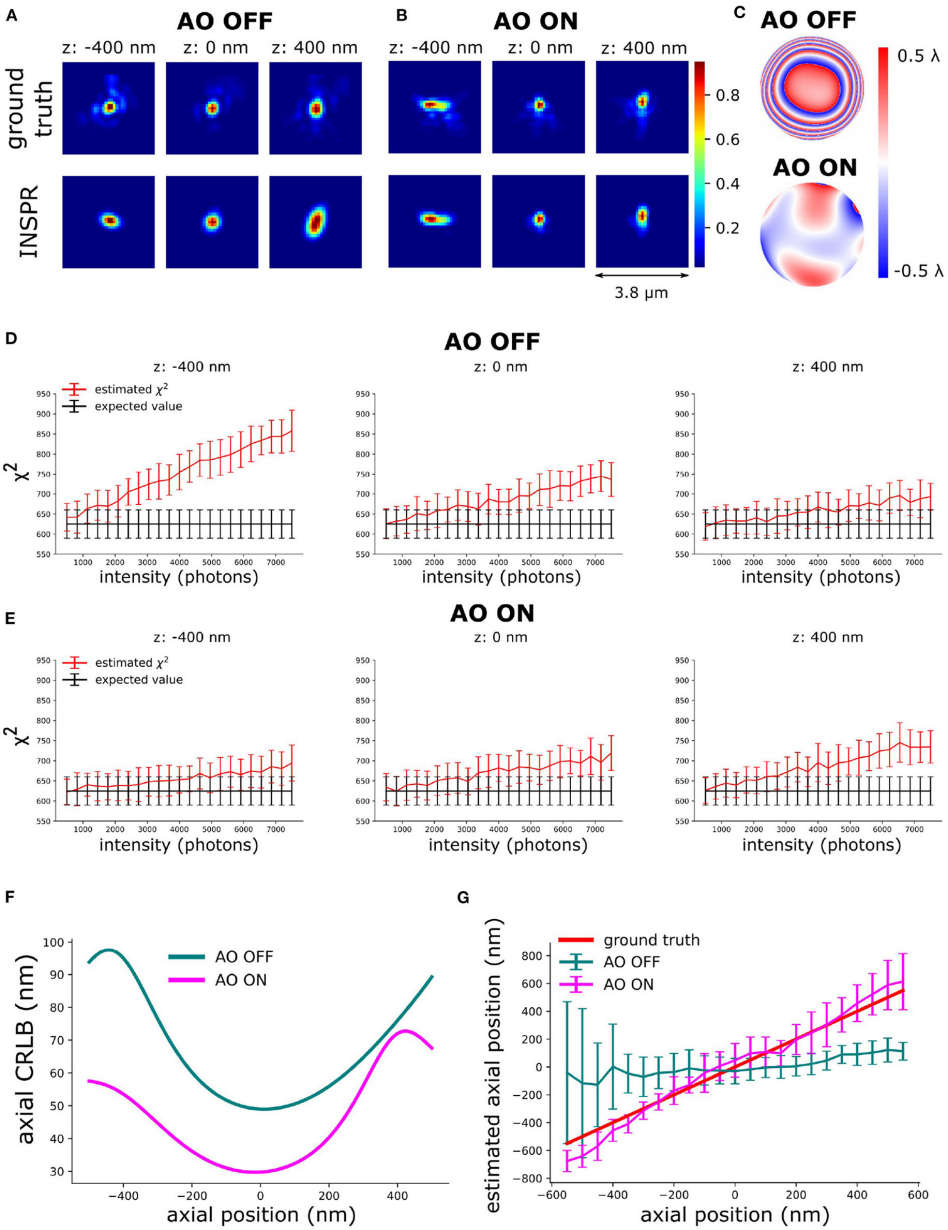
Validation of the principle of AO-SOLEIL. **(A)** PSFs without AO correction. **(B)** PSFs with AO correction. **(C)** Pupil phase of ground truth PSF with addition 100 mλ astigmatism for 3D SMLM. **(D)** χ^2^ value of AO OFF PSF at different intensity and depth (–400, 0, and 400 nm). In this test, we fixed the I/bg ratio to 33. **(E)** χ^2^ value of AO ON PSF at different intensity and depth (–400, 0, and 400 nm). In this test, we fixed the I/bg ratio to 33. **(F)** Curve of axial CRLB vs. axial position (intensity: 1,000 photons, background: 30 photons/pixel). **(G)** Curve of axial position vs. estimated axial position. For each data point, we repeated the localization 51 times. The errorbar is the standard deviation of estimated axial position.

**Figure 5:**
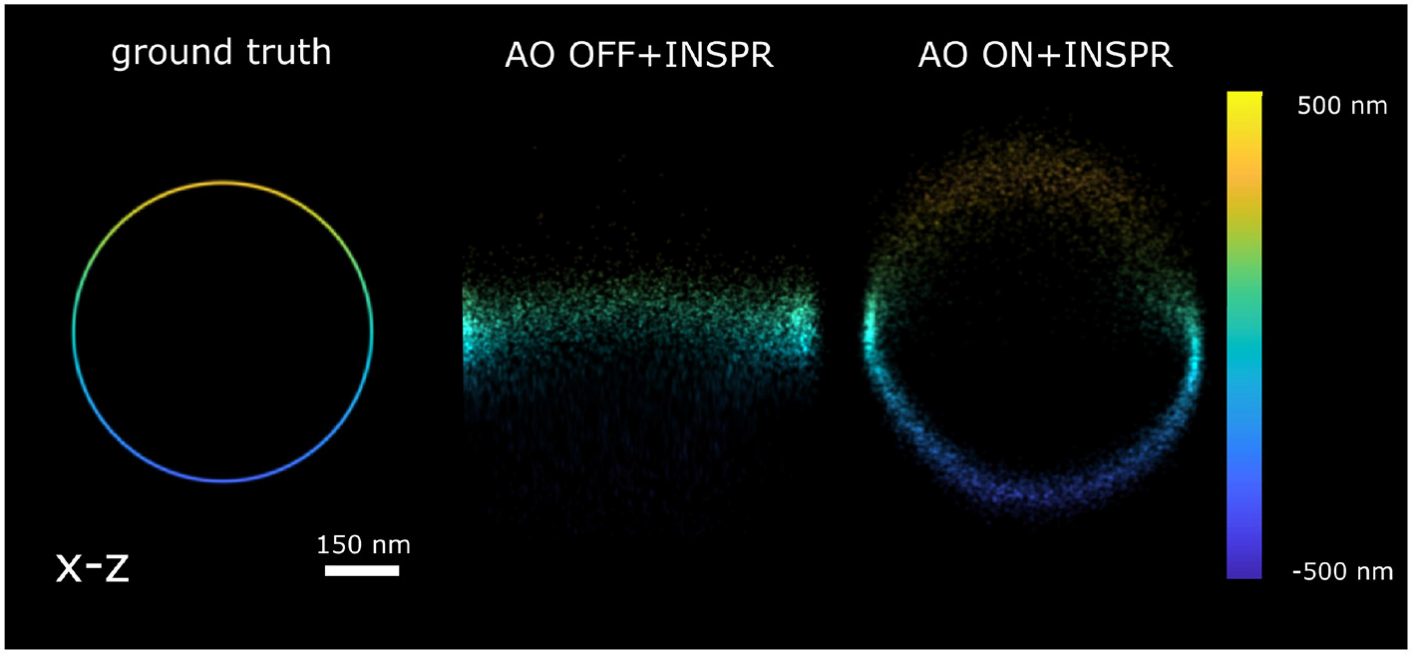
Reconstruction x-z cross-section of the 3D ring localized by AO OFF and AO ON INSPR PSF model. ground truth: the x-z view of 3D ring. AO OFF+INSPR: the reconstruction image without AO correction, but *in-situ* PSF estimated using the INSPR algorithm. AO ON+INSPR: the reconstruction image with AO and the *in-situ* PSF estimated using the INSPR algorithm.

**Figure 6:**
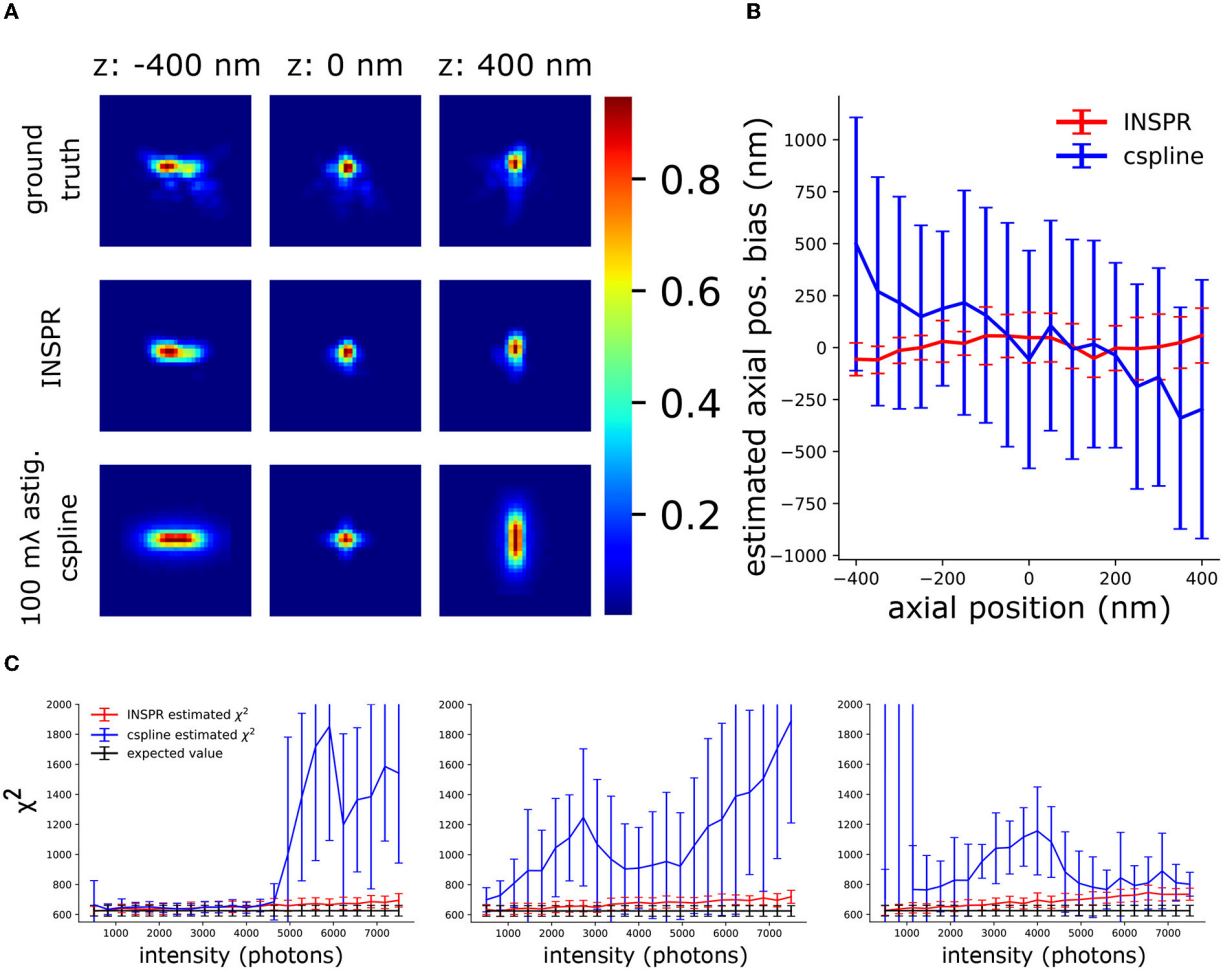
Investigation of the synergy between INSPR and AO-SOLEIL. **(A)** PSFs with AO correction. Ground truth: The PSF simulated using the true Zernike aberrations. INSPR: PSFs calibrated from the raw SMLM data. 100 mλ astig. cspline: cubic spline PSF model calibrated by z-stack PSFs data without sample-induced aberration. **(B)** The axial position bias (*z*_*true*_−*z*_*estimated*_). **(C)** χ^2^ of PSF models estimated using INSPR and cspline calibration.

We used active aberration correction to correct the first 7 Zernike modes. Subsequently, *in-situ* PSF calibration (INSPR) was used to calibrate the 3D PSF model (INSPR PSF). To analyze the synergy between aberration correction and *in-situ* PSF calibration, we performed *in-silico* experiments (Figures 4–6). We used the SDS simulator (details of SDS simulator is in Supplementary Section 2) mentioned above to simulate blinking images in the presence of aberrations. To mimic the high aberration situation, we simulated PSFs (see Section 7) by randomly assigning 150 mλ Zernike aberrations uniformly distributed over the first 17 Zernike modes ($Z_{2}^{\pm 2}$,$Z_{3}^{\pm 3,\pm 1}$,$Z_{4}^{\pm 4,\pm 2,0}$, $Z_{5}^{\pm 5,\pm 3,\pm 1}$) excluding the piston, tip, tilt, and defocus aberrations. Spherical aberration (220 mλ) was added to mimic the refractive index mismatch between oil immersion and tissue. We term this set of Zernike aberrations as the initial i.e. AO OFF aberration. Then, combining with the SDS, we used the proposed sensorless AO algorithm to correct the 7 Zernike modes ($Z_{2}^{\pm 2}$, $Z_{3}^{\pm 1}$, $Z_{3}^{\pm 3}$, $Z_{4}^{0}$). In this step, we input the Zernike aberrations into the SDS and generated images with aberrated blinking single-molecules. Based on this, we can investigate the performance of the proposed sensorless AO algorithm *in-silico*.

To enable the 3D localization of single-molecules an astigmatic PSF was chosen. This PSF was created by adding 100 mλ astigmatism aberration ($Z_{2}^{2}$). To estimate the *in-situ* PSF, we simulated 1,000 frames of blinking images (intensity: 2,000 photons, background: 30 photons/pixel) with the initial aberration (AO OFF) and the final aberration after correction (AO ON) (Figures 4A,B). The ground truth PSFs were computed based on the true initial (AO OFF) and final aberration (AO ON) (Figure 4C).

The χ^2^ value was used to compare and evaluate the accuracy of the PSF models (Siemons et al., 2018).


(7) $$\begin{array}{ll} \chi^{2} & =\overset{K}{\underset{i=1}{\sum }}\frac{{\left(n_{i}-u_{i}\left(\hat{\theta }\right)\right)}^{2}}{u_{i}} \end{array}$$

(8) $$\begin{array}{ll} u_{i}\left(\hat{\theta }\right) & ={\hat{\theta }}_{I}\cdot PSF_{i}\left({\hat{\theta }}_{x},{\hat{\theta }}_{y},{\hat{\theta }}_{z}\right)+{\hat{\theta }}_{bg} \end{array}$$

where K is the number of pixels of the region of interest, *n*_*i*_ is the photon count of the data at the i^*th*^ pixel, *u*_*i*_ is the photon count in i^*th*^ pixel, $PSF_{i}\left({\hat{\theta }}_{x},\theta_{y},{\hat{\theta }}_{z}\right)$ is the 3D PSF model in i^*th*^ pixel, $\hat{\theta }$ is the vector with the estimates containing the estimands θ_*x*_, θ_*y*_, θ_*z*_, θ_*I*_, θ_*bg*_. These correspond to the x,y,z position, emitter intensity, and the emitter background, respectively. To calculate $u_{i}\left(\hat{\theta }\right)$, the $\hat{\theta }$ is obtained using maximum likelihood estimation (Smith and Joseph, 2010).

For Poissonian distributed measurement the expected χ^2^ value (*E*[χ^2^]) and the variance of χ^2^ value (*var*[χ^2^]) can be expressed as following:


(9) $$\begin{array}{ll} E\left[\chi^{2}\right] & =K \end{array}$$

(10) $$\begin{array}{ll} var\left[\chi^{2}\right] & =2K+\overset{K}{\underset{i=1}{\sum }}\frac{1}{u_{i}} \end{array}$$

The above equation delivers a statistical way to determine whether the PSF model is accurate and when it's not. The PSF is statistically different (not accurate) from the data when the χ^2^ value is larger than the expected χ^2^ value plus its standard deviation. The χ^2^ was computed based on Equation (8). In Figures 4D,E, the expected χ^2^ value of the estimated PSF models vs. intensity are shown. The curve of estimated PSF models are computed by keeping the estimated PSF model constant and generating 51 independent noise realizations (Equation 8). We observed that the χ^2^ value increases with increasing intensity (Figures 4D,E). We observed that the INSPR PSF estimated model without AO was statistically different at 1500 photons. The INSPR PSF estimated model with AO was statistically different until 3,000 photons.

In Figure 4F, we calculated the theoretical axial CRLB over different axial positions and we observed the improvement of theoretical axial CRLB with AO correction over without AO correction. We observed a 200% improvement of the axial CRLB. Furthermore, the impact of a model mismatch between the estimated and the ground truth PSF was analyzed by calculating the estimation bias in the z direction (Figure 4G). To do this we generated the raw SMLM images with 1,000 photons and background 30 photons/pixel. The reason for choosing 1,000 photons intensity is to make sure the INSPR model is not statistically different from ground truth PSF model and 30 photons/pixel background is to match with the I/bg ratio of 33.33 in χ^2^-test (Figure 4). The INSPR PSF model obtained from INSPR was used for the localization. Figure 4G shows the estimated axial position vs. the ground truth axial position. We observed that the estimated axial position is strongly biased without AO. With AO correction the bias is significantly reduced. The ground z position is within the standard deviation of estimated axial position up to ±400 nm. To visualize the impact of the improvement of the CRLB and reduction of the bias raw SMLM data of a 3D ring was simulated. In the 3D ring test, we simulated the 3D PSF blinking spots along the 3D ring structure with the ground truth PSF models and used INSPR PSF model for localization. The reconstructions are shown in Figure 5. We found that in the AO OFF situation the reconstruction has completely failed. This result aligns with the observation in Figure 4G, which is the strong axial position bias occurs in AO OFF model. This result again justifies our motivation to combine AO correction and INSPR mehtod.

To analyze the use of *in-situ* PSF calibration and not use the pre-calibrated cubic spline (cspline) PSF (Babcock and Zhuang, 2017; Li et al., 2018), we performed an in-slico experiment (Figure 6). In this experiment, we have the PSFs after AO correction in Figure 4B and we also have the PSF model calibrated by INSPR. In addition, to mimic a pre-calibrated PSF model from a thin sample with fluorescence beads we simulated astigmatism PSF only with 100 mλ astigmatism aberration ($Z_{2}^{2}$) and without other aberrations and did a cspline calibration with Super-resolution Microscopy Analysis Platform (SMAP, EMBL Heidelberg) (Ries, 2020) (Figure 6A). In Figure 6B, we show the axial localization bias using INSPR PSF model and cspline PSF model. We observed a strong axial bias from the cspline PSF model. In Figure 6C, we show the χ^2^ value of cspline and INSPR model, which suggests that a pre-calibrated cspline method is not accurate in tissue SMLM imaging.

## 3. Methods

### 3.1. Initial aberration correction of the system

To compensate for the aberrations introduced by the DM and the static aberrations of the microscope we acquired PSFs by imaging single 23 nm beads embedded in 1% agarose. Here, because our input image is small and contains only a single PSF, we don't need to use the extended image quality metric from the previous section, but can just minimize the mean width of the PSF by adopting a second moment metric function:


(11) $$\begin{array}{l} {\text{M}}_{\text{sec}}=\overset{N}{\underset{i=1}{\sum }}\overset{N}{\underset{j=1}{\sum }}\text{I}\left(i,j\right)\cdot \left[{\left(i-c_{x}\right)}^{2}+{\left(j-c_{y}\right)}^{2}\right]/\overset{N}{\underset{i=1}{\sum }}\overset{N}{\underset{j=1}{\sum }}\text{I}\left(i,j\right), \end{array}$$

where M_sec_ is the second moment of single PSF, I(*i, j*) is the pixel value at row *i* and column *j* of the acquired image, *c*_*x*_ and *c*_*y*_ are the center of mass of I(*i, j*), and *N* is both the width and height of the camera image in pixels. In our experiment, we chose *N* as 21 pixels. The metric is minimized by use of the adaptive Nelder-Mead algorithm from the Scipy library (version 1.7.3) for 3,000 iterations with 30 ms of exposure time for each frame. This minimization is not performed in the Zernike basis, but in that of the deformable mirror control voltages, because it leads to more robust performance when strong aberration present in the system (Siemons et al., 2021). In principle, maximum intensity or sharpness metric functions can also be adopted as metric function to minimize the system aberration (Olivier et al., 2009; Linhai and Rao, 2011). However, these metric functions are more susceptible to the photo-bleaching making they unsuited for our application.

### 3.2. Data acquisition for sensorless AO-SOLEIL microscopy

To reduce the imaging background the sample was pre-bleached for 30–60 s. The pre-bleaching was done while moving the piezo stage (Smartact; x,y an SLC1730; z an SLC1720) through the targeted axial area. Sensorless AO was subsequently performed on this desired 3D FOV while illuminating the sample with widefield illumination (integration time 30 ms).

After AO correction, first the widefield SMLM acquisition was finished and subsequently the SOLEIL benchmark. This order may potentially decrease the quality of SOLEIL imaging including the estimated CRLB and number of localizations per frame because of photo-bleaching. However, in all imaging, we still observe the improvement of SOLEIL microscopy over widefield microscopy. The SOLEIL acquisition deviates from the previous published work (Hung et al., 2022). In this work, the galvo mirror continuously translated the SOLEIL illumination from the top to the bottom of the FOV. To enable a virtual confocal readout (Baumgart and Kubitscheck, 2012; Chakraborty et al., 2020) we used a rolling shutter by activating the Andor SOLIS LightScan PLUS function, see Supplementary Section 9. This allowed us to synchronize the rolling shutter readout of the camera to be synchronized with the light sheet illumination. To synchronize the rolling shutter and SOLEIL illumination, the camera trigger mode was set to external trigger and the camera and galvo mirror were controlled by an Arduino micro-controller.

### 3.3. Cell culture

#### 3.3.1. Caco2-BBE cells for artificial multilayer sample

Caco2-BBE cells (a gift from S.C.D. van IJzendoorn, University Medical Center Groningen, the Netherlands) were maintained in DMEM supplemented with 9% FBS (fetal bovine serum), 50 μg/μl penicillin/streptomycin and 2 mM L-glutamine at 37°C and 5% CO_2_. Cells were seeded on 18 mm coverslips at a density of 1·10^5^/cm^2^ and cultured for 10–12 days to allow for spontaneous polarization and brush border formation. The monolayer of cells was fixed with 4% paraformaldehyde (PFA) in phosphate-buffered saline (PBS) for 10 min, washed with PBS (3 × 5 min), permeabilized with 0.5% Triton X-100 in miliQ water for 15 min, washed with PBS (3 × 5 min) and blocked with 3% BSA in PBS for at least 1 h. Cells were incubated overnight at 4°C with a primary antibody against ezrin (mouse, BD Biosciences, 610602, dilution 1:500) from now referred to as ezrin labeled cells. After washing in PBS (3 × 5 min), the cells were incubated with secondary antibody [goat, anti-Mouse IgG (H+L), Alexa Fluor 647 (Life Technologies, dilution 1:500)] for 1 h at room temperature (RT) and washed with PBS.

#### 3.3.2. Caco2-BBE cells for spheroid sample

To create a more three-dimensional culture a monolayer of Caco2-BBE cells, similar as is mentioned above, was perturbed by forcibly pipetting the culturing medium over the cells. The resulting cell clumps were cultured in suspension for an additional 3 days to allow for the formation of spheroid like structures. Spheroids were fixed with 4% PFA and 4% sucrose in PBS for 15 min, washed with PBS (3 × 5 min), permeabilized with 0.5% Triton X-100 in miliQ water for 30 min, washed with PBS (3 × 5 min) and blocked with 3% BSA in PBS for at least 1 h. Cells were incubated overnight at 4°C with a primary antibody against cytochrome C (mouse, BD Biosciences, 556432, dilution 1:500) from now on referred to as mitochondria labeled cells. After washing in PBS (3 × 5 min), the cells were incubated with secondary antibody [goat, anti-Mouse IgG (H+L), Alexa Fluor 647 (Life Technologies, dilution 1:500)] for 3 h at RT and washed with PBS.

#### 3.3.3. Preparation of *Drosophila* brains

Flies (*Drosophila melanogaster*) were kept on Nutri-Fly Bloomington Formulation with dry yeast at 20°C. Males and females were used as no gender-specific differences were observed. Genotypes used: *10xUAS-IVS-mCD8::GFP* / +; *MB077c-Gal4* / + (control) and *10xUAS-IVS-mCD8::GFP* / *10xUAS-myr::4xSNAPf* ; *MB077c-Gal4* / + (experiment). Adult flies were aged at 20°C for 5–7 days before performing brain dissection (Paglione et al., 2020). Brain dissections were performed as described (Paglione et al., 2020). Briefly, decapitated heads were fixed in 4% formaldehyde (47608-250ML-F, Sigma-Aldrich) in PTX (0.1% Triton X-100 (T9284, Sigma-Aldrich) in PBS) for 20 min, and washed 3 × 10 min with PTX. Brain dissections were performed in PTX, and dissected brains were fixed in 4% formaldehyde in PTX for 10 min, followed by 3 × 10 min washes with PTX. Brains were incubated with SNAP-Surface Alexa Fluor 647 (Inc. S9136S, New England Biolabs) at a concentration of 0.0625 μM with rotation for 15 min at RT. Brains were washed 3 × 10 min with PTX, and subsequently PTX was completely removed and 200 μL PBS was added.

### 3.4. Sample preparation and mounting

#### 3.4.1. Preparation of the large fluorescence bead sample

We prepared a glass slide with four strips of double sided tape arranged on the four sides of a rectangle. After that, 1% agarose solution was prepared by adding 100 mg of agarose powder (BP160-100, Thermo Fisher Scientific, Waltham MA, U.S.A.) to 10 mL of PBS buffer followed by 20 min stirring with a magnetic stirrer at 100°C. Then, we prepared a 1:10,000 diluted fluorescence bead stock (Tetraspeck, Thermo Fisher Scientific) with 1% agarose solution. We quickly mixed the diluted fluorescence bead stock with the agarose solution and added 200 μL of mixture in the middle of double sided tape. Before the agarose gel became solid, a #1.5 coverslip was mounted on the glass slide attaching the double sided tape. Then, we used nail polish to seal the coverslip. The final thickness of the sample is about 120 μm.

#### 3.4.2. Spheroid Caco2-BBE cell dSTORM sample

**Figure 7:**
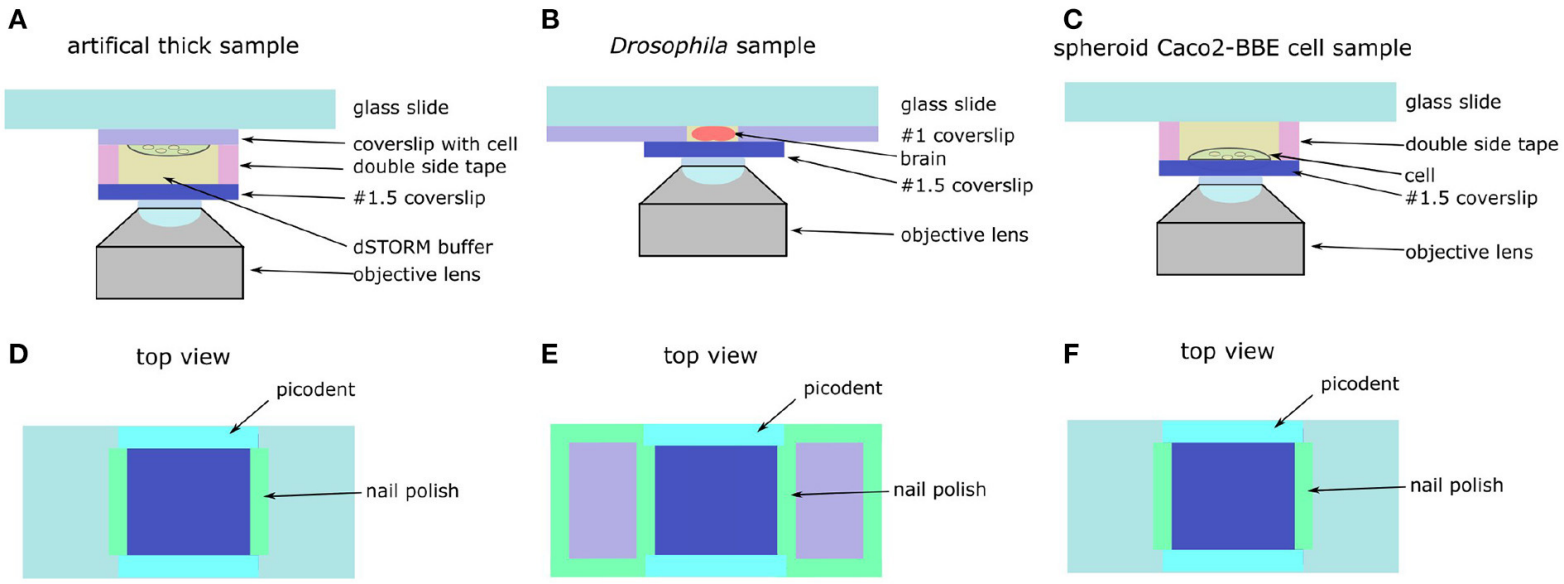
schematic of artificial thick and *Drosophila* brain. **(A)** Side view of artificial thick sample. **(B)** Side view of *Drosophila* brain. **(C)** Side view of spheroid Caco2-BBE cell sample. **(D)** Top view of artificial thick sample. **(E)** Top view of *Drosophila* brain. **(F)** Top view of spheroid Caco2-BBE cell sample.

Firstly, we put two strips of double sided tape on a glass slide (MS10UW, Thorlabs) as spacer (Figure 7C). The spheroid Cao2-BBE cell was stored in PBS in Eppendorf tube. We picked up the spheroid Caco2-BBE cell from the Eppendorf tube by pipette and put the cell on a #1.5 coverslip (CG00C2, Thorlabs). Then, 20 μl of 1% agarose (BP160-100, Thermo Fisher Scientific, Waltham MA, U.S.A.) diluted by PBS was added to stabilize the cell. Before the agarose became solid, the coverslip with the cell was mounted on a glass slide attached with the double sided tape. Then, we used nail polish to seal the edge between the coverslip and the double sided tape. After the nail polish was dry, we added the dSTORM buffer into the chamber and used two-component gel (Picodent, Wipperfürth) to seal the front and backside of the coverslip (Figure 7F).

#### 3.4.3. Artificial Caco2-BBE cell dSTORM sample

A coverslip with Caco2-BBE cell adhered on the microscopy glass slides with double sided tape (Figure 7A). Another coverslip was attached to the cell coverslip with 65 μm thick double sided tape (details of sample mounting is in Supplementary Figure S3). The thickness of double sided tape was measured by using a homemade setup (Supplementary Figure S4). The left and right sides of the coverslip were sealed by using nail polish and the front and backside of the coverslip were sealed by using the two-component gel. Compared with the original sample, the artificial Caco2-BBE sample had an additional 65 μm deep buffer layer, which introduced an additional 0.38 λ spherical aberration estimated based on the equation in Booth and Wilson (2001).

#### 3.4.4. Fluorescently labeled single neurons in *Drosophila* brains for dSTORM imaging

*Drosophila* brains were mounted in glass slides as previously described in Kelly et al. (2017). The dissected brains were stored in PBS buffer at 4°C in 1.5 mL tubes until ready to be mounted. Then, we glued two #0 coverslips (CG00C2, Thorlabs) on a glass slide (MS10UW, Thorlabs) by using nail polish (Figure 7E). The distance between the two coverslips was around 5 mm. A pipette was used to pick up a brain from Eppendorf tube and put the brain on the glass slide between the two #0 coverslips. Then, the brain was placed in the correct orientation with the help of a 20X magnification (LCAch N 20X, Olympus) stereo microscope (NAME NUMBER). Then, we added 10 μl of 1% agarose diluted in PBS on the brain. Before the agarose became solid, a #1.5 coverslip was mounted on the brain and attached to the #1 coverslips. The left and right sides of the coverslip were sealed with nail polish and the front and backside were sealed by using two-component gel (Figure 7D).

#### 3.4.5. Preparation of dSTORM buffer

In this research, we used oxygen scavenger buffer (Glox-buffer). We prepared a glucose stock solution (300 mM glucose, 50 mM Tris, 10 mM NaCl dissolved in Milli-Q H_2_O) and stored it at 4°. The final concentration of each ingredients are 1.25 mg/ml catalase (Sigma, C40-100MG), 1 mg/ml glucose-oxidase (Sigma, G2133-10KU), and 50~150 mM MEA (Sigma, 30070-10G) diluted in glucose stock. We adjusted the blinking density by adjusting the concentration of MEA.

### 3.5. SMLM data analysis

To perform 2D localization, drift correction, filtering, and visualization, we used Super-resolution Microscopy Analysis Platform (SMAP) (Ries, 2020). We used maximum likelihood estimation (MLE) with a 2D Gaussian PSF model for the estimation. The estimands are the position, intensity, background counts, and the width of the Gaussian PSF model.

To perform 3D PSF calibration, localization, and drift correction, we used the INSPR algorithm (Xu et al., 2020) to build the PSF and to localize the single molecules. The region of interest (ROI) of the PSF was 27 pixels which correspond to 2.92 μm. To calibrate the PSF with INSPR only the first 36 Zernike basis functions were considered. The 3D reconstruction was made with SMAP.

## 4. Results

### 4.1. Impact of spherical aberration on the three-dimensional localization precision

**Figure 8:**
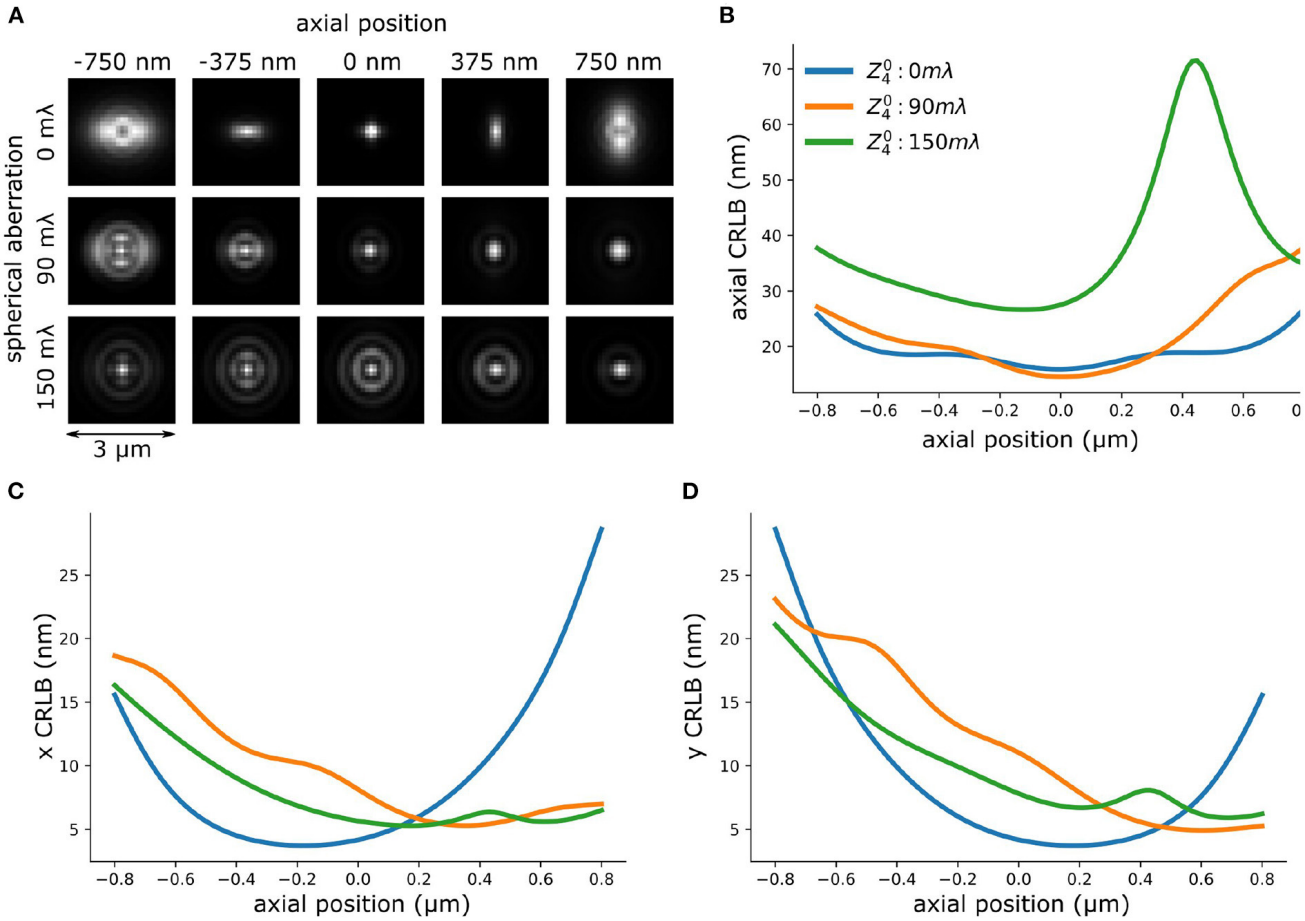
Spherical aberration deteriorates axial CRLB of astigmatism-based PSF. **(A)** PSFs with different amplitude of spherical aberration. **(B)** Theoretical axial CRLB of INSPR PSF models with different amplitude of spherical aberration. **(C,D)** Theoretical lateral (x,y) CRLB of INSPR model with different amplitude of spherical aberration.

To investigate the performance of the INSPR algorithm when spherical aberration presents in the PSF model we performed a set of *in-silico* experiments. We simulated vectorial PSFs with varying spherical aberrations (0 ~ 150 mλ) (Figure 8A) (Siemons et al., 2018). The details of the PSF simulation is in Supplementary Section 2. Then, we used the INSPR algorithm to calibrate the PSF model. The *in-situ* calibrated PSF model was used for computing the CRLB. We observed that an increased spherical aberration gradually reduces the ellipticity of astigmatism-based PSF gradually, which reduces the ability to localize in three dimensions. This is inline with previous reported observations (Siemons et al., 2020). We observed in simulation that spherical aberration deteriorates the axial CRLB (Figure 8B), but not the lateral CRLB (Figures 8C,D). This *in-silico* observation aligns with our experimental observation (Figure 8 and Supplementary Figure S2). To investigate if this observation is dependent on the algorithm that was used for PSF calibration, we repeated the same simulation using cubic splines (Supplementary Figure S5) (Babcock and Zhuang, 2017; Li et al., 2018). We observed a deteriorated axial CRLB when spherical aberration presents in the PSF.

### 4.2. Sensorless AO performance and benchmark

In this section, we analyze and benchmark the performance of sensorless AO algorithms *in-silico* based on SDS simulator (details of SDS simulator is in Supplementary Section 2).

**Figure 9:**
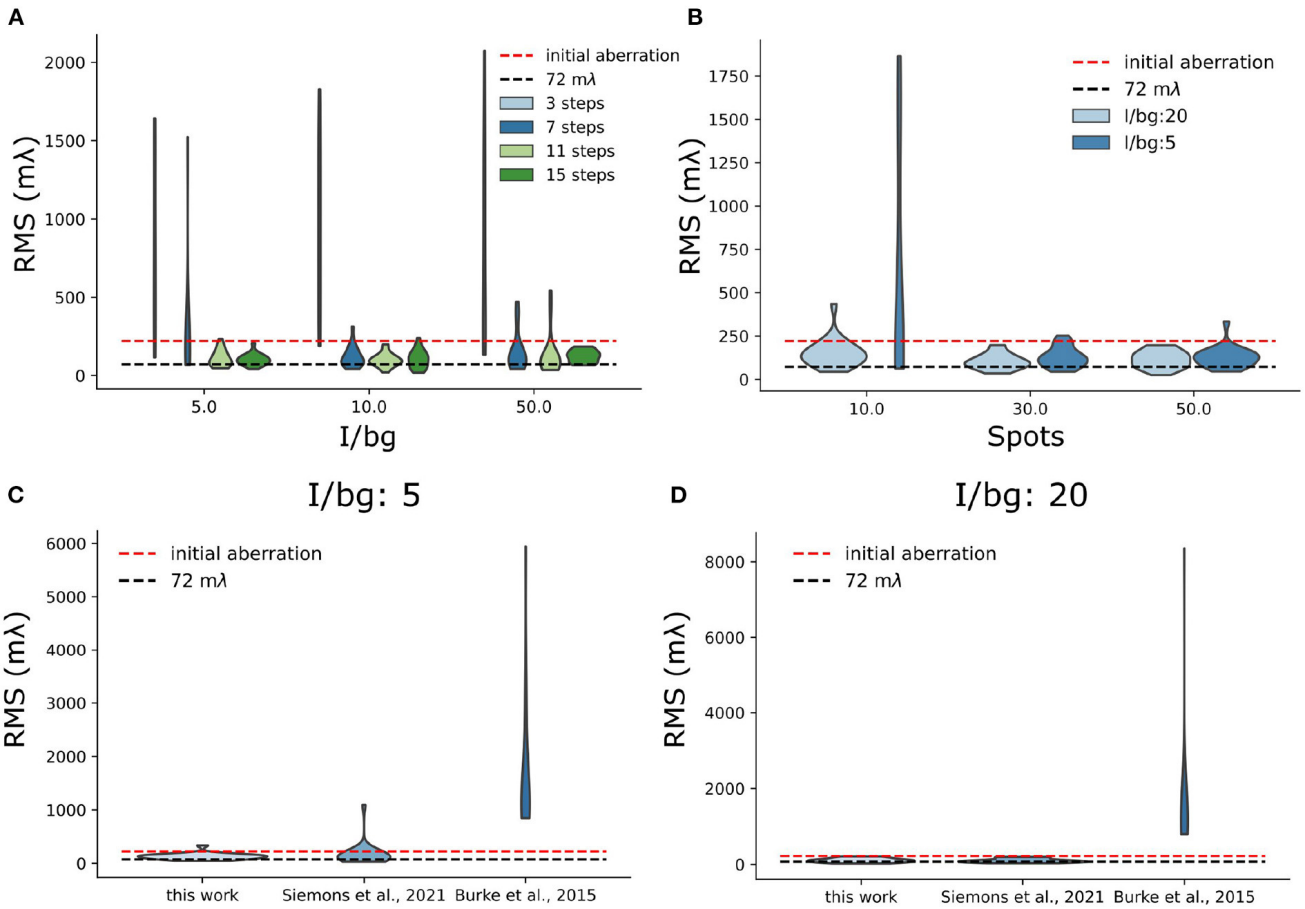
Simulation test of sensorless AO algorithm. **(A)** Stability test of sensorless regarding the number of measurement step per frame. **(B)** Stability test of sensorless regarding the number of blinking single-molecules per frame. For this test, we used 11 measurement steps. **(C,D)** Benchmark different sensorless AO correction metric functions under different I/bg ratio (I/bg:5 and I/bg: 20).

In the *in-silico* experiments, we focused on two aspects of sensorless AO algorithm: the number of measurements needed and the sparsity of the acquired images. We found that the number of measurement steps is important for a robust sensorless AO correction. In previous research (Debarre et al., 2007), it was found that in theory 3 measurement steps for correcting a Zernike mode are sufficient. Less measurements can be expected by using more advanced algorithms (Booth, 2006). However, in Figure 9A, we found that 7 measurement steps are the minimal number of measurement steps for stable AO correction in low background situations (I/bg is 10 and 50). We think the reason is that the sparse blinking of localization microscopy images delivers a weak signal in Fourier space, which increases the noise in OTF-based metric functions. For high background situations (I/bg ratio is 5), 11 steps can be more stable than 7 steps. Nevertheless, we didn't observe significant improvement between the result between 11 and 15 measurement steps.

We also observed that the stability of the sensorless AO algorithm depends on the number of blinking single-molecules. In Figure 9B, we investigated the sensorless AO algorithm with different number of blinking spots per frame (10, 30, and 50). The size of each frame is 30 μm × 30 μm. The pixel size is 108.33 nm, which is same as our system. In this experiment, we chose the I/bg ratio as 20 and the intensity of each blinking single molecules as 2,000 photons. The initial aberrations were uniformly assigned to the 7 Zernike modes, which is the number of modes that we correct in this research. We scaled the initial aberration to be 220 mλ (RMS value). For AO correction we used 11 measurement steps. To investigate the robustness of the approach the experiment was repeated 20 times and we tested low and high background situations corresponding to the I/bg is 20 and 5, respectively. In Figure 9B, we observed that when the number of blinking single-molecules is lower than 30 single-molecules/frame, the sensorless AO correction is likely to become unstable. The high background situation can further deteriorate the result (Figure 9B). We didn't observe significant improvement of the result between 30 single-molecules per frame and 50 single-molecules per frame. In the real experiment, we control the concentration of MEA to adjust the number of blinking. However, it should be noted that in SMLM there is a limit to the number of single-molecules that can be on, because the sparsity of single-molecule is used to achieve the resolution improvement in SMLM.

In addition, we also benchmark our metric with other metric functions used in localization microscopy (Burke et al., 2015; Siemons et al., 2021) (Figures 9C,D). We found comparing with other metric functions, the proposed metric in this work is more stable in high background situation (I/bg: 5), which suggests it is more stable when performing sensorless AO in tissue imaging (Figure 9C). Nevertheless, in low background situation (I/bg: 20), we didn't observe improvement over REALM (Siemons et al., 2021) (Figure 9D).

### 4.3. 2D SMLM of Erzin in an artificial thick sample with Caco2-BBE cells

**Figure 10:**
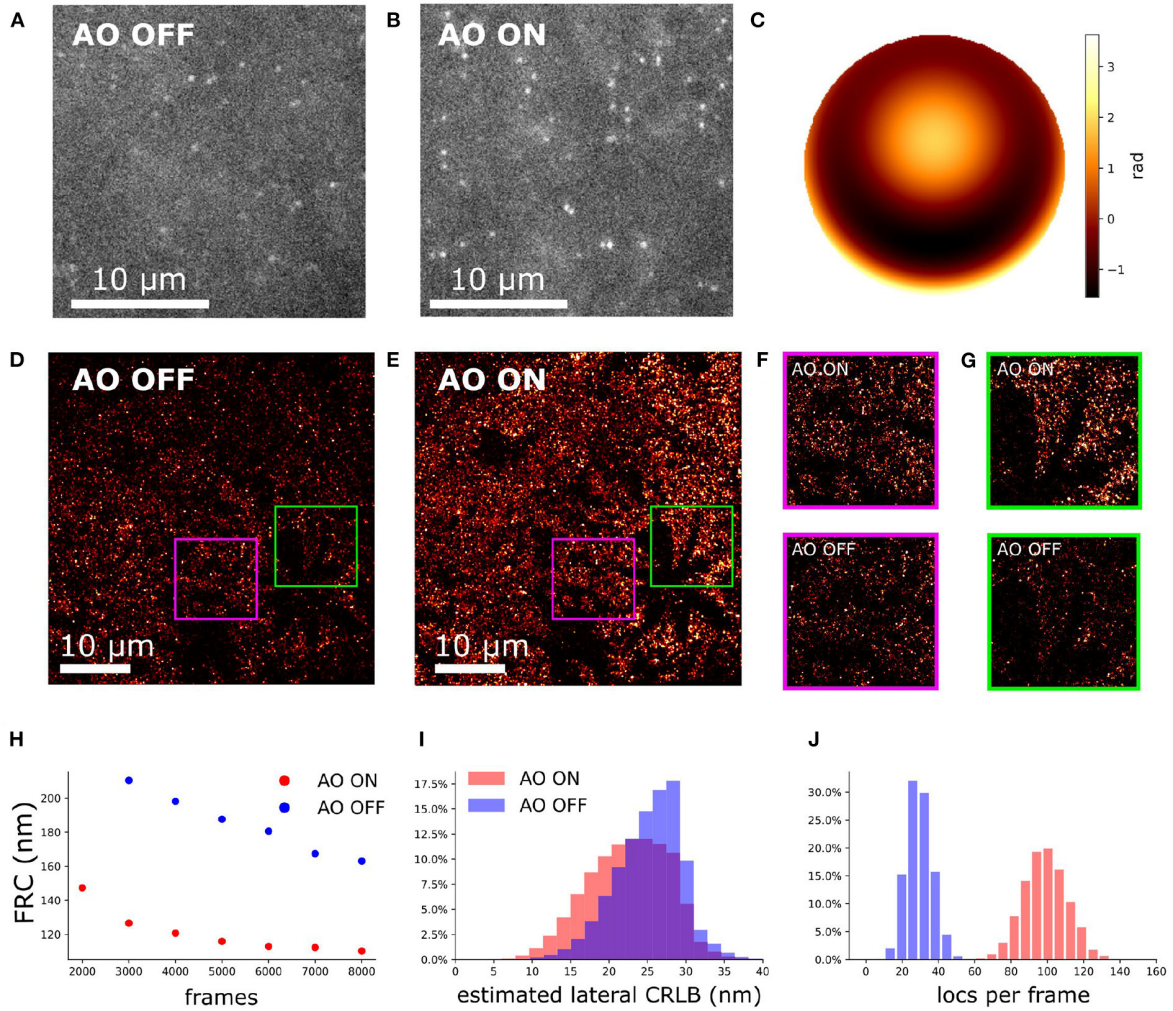
Aberration correction on an artificial thick sample with Caco2-BBE cells labeled against Ezrin. **(A)** Raw camera image of Caco2-BBE STORM sample without aberration correction. **(B)** Raw camera image of Caco2-BBE STORM data with aberration correction. **(C)** The pupil phase for aberration correction. **(D)** Reconstruction image of Caco2-BBE sample without aberration correction. **(E)** Reconstruction image of Caco2-BBE sample with aberration correction. **(D,E)** Rendered with the same contrast to visualize the localization density. **(F)** Zoom-in of **(D,E)**. **(G,H)** FRC of **(D,E)** verse the frame number. **(I)** Lateral CRLB distribution from the data-set of **(D,E)**. **(J)** Number of detected localizations per frame from the dataset of **(D,E)**.

To investigate the performance of our AO in a controlled environment we created an artificial thick sample. To create a predominantly spherical aberration we added a 65 μm thick STORM buffer layer to the Caco2-BBE cell. The Caco2-cells with labeled Erzin to perform dSTORM was at the top of the sandwich (Supplementary Figure S3). This design introduced an additional 380 mλ spherical aberration. The correction enhances the sharpness of PSF (Figures 10A,B) and improved the estimated localization precision estimation (Figure 10I) and detection efficiency (Figure 10J). The correction phase is shown Figure 10C. This hypothesis was confirmed by simulation (Supplementary Figure S6). The corresponding reconstructions show a better contrast with correction than without correction (Figures 10D–G). A quantitative comparison of the localizations shows a three times higher detection efficiency (Figure 10J), a 12% improvement in CRLB (Figure 10I) and a 45% improvement in Fourier Ring Correlation (FRC) (Figure 10H) (Nieuwenhuizen et al., 2013).

### 4.4. 2D SMLM of mitochondria in spheroid Caco2-BBE cells

**Figure 11:**
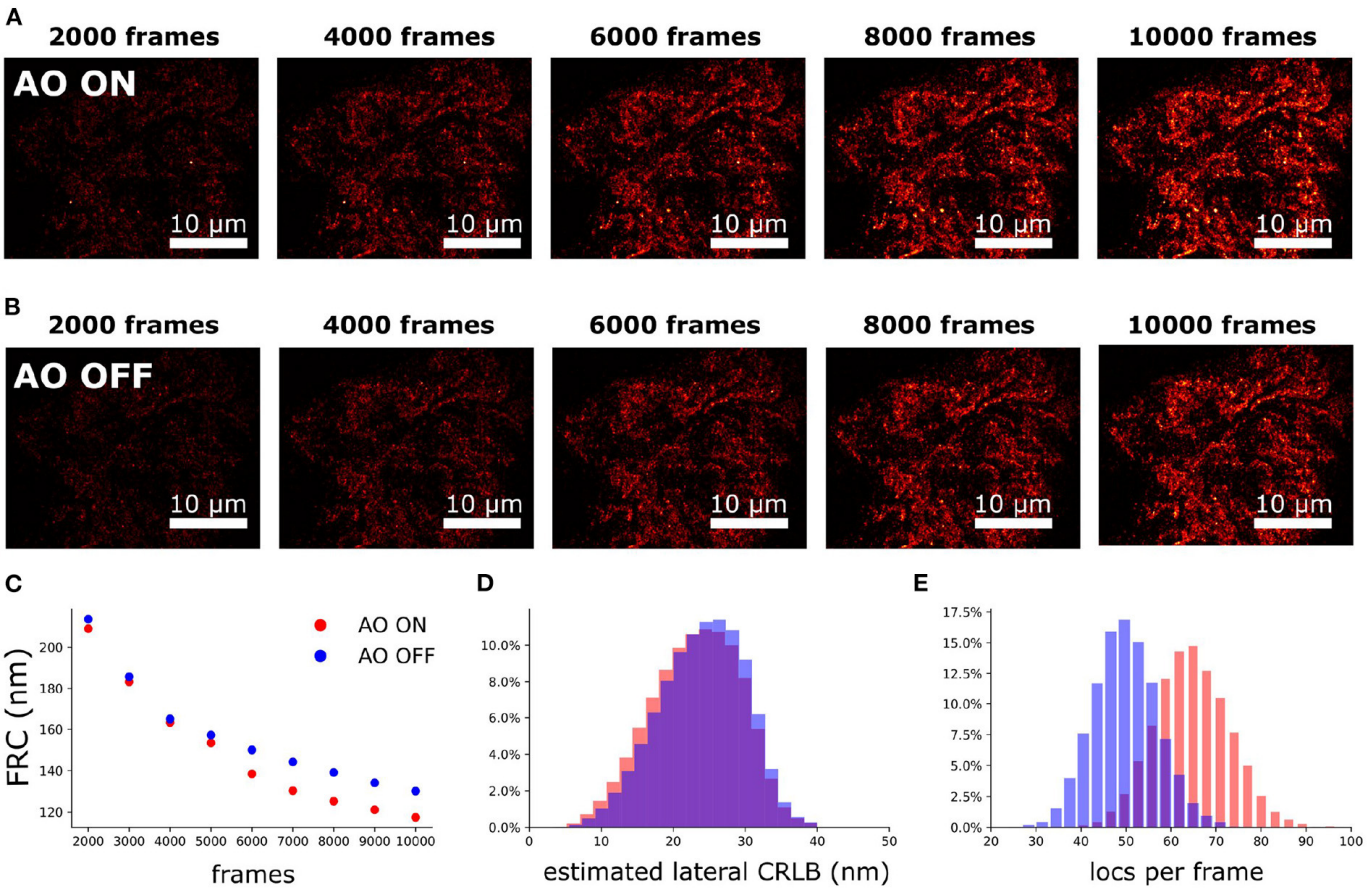
Aberration correction on mitochondria of spheroid Caco2-BBE cells (depth is 17 μm). **(A)** SMLM reconstruction with correction. **(B)** SMLM reconstruction without correction. **(A,B)** Were rendered with same contrast to visualize the localization density. **(C)** FRC of **(A,B)** vs. the frame number. **(D)** Lateral CRLB distribution from the data-set of **(A,B)**. **(E)** Number of detected localizations per frame from the dataset of **(A,B)**.

To investigate the performance of the sample-induced aberration correction algorithm we imaged spheroid Caco2-BBE cells at a depth of 17 μm using widefield microscopy (exposure time of 30 ms). The reconstruction images with and without AO correction are shown in Figures 11A,B. For 2D SMLM we observed that the spot detection efficiency was improved by 30% with correction (Figure 11E) and ultimately the FRC resolution (Nieuwenhuizen et al., 2013) was improved by 11% (Figure 11C). The median estimated lateral CRLB is improved by 1 nm with correction (Figure 11D).

### 4.5. 2D SMLM of a single neuron in an adult *Drosophila* brain

**Figure 12:**
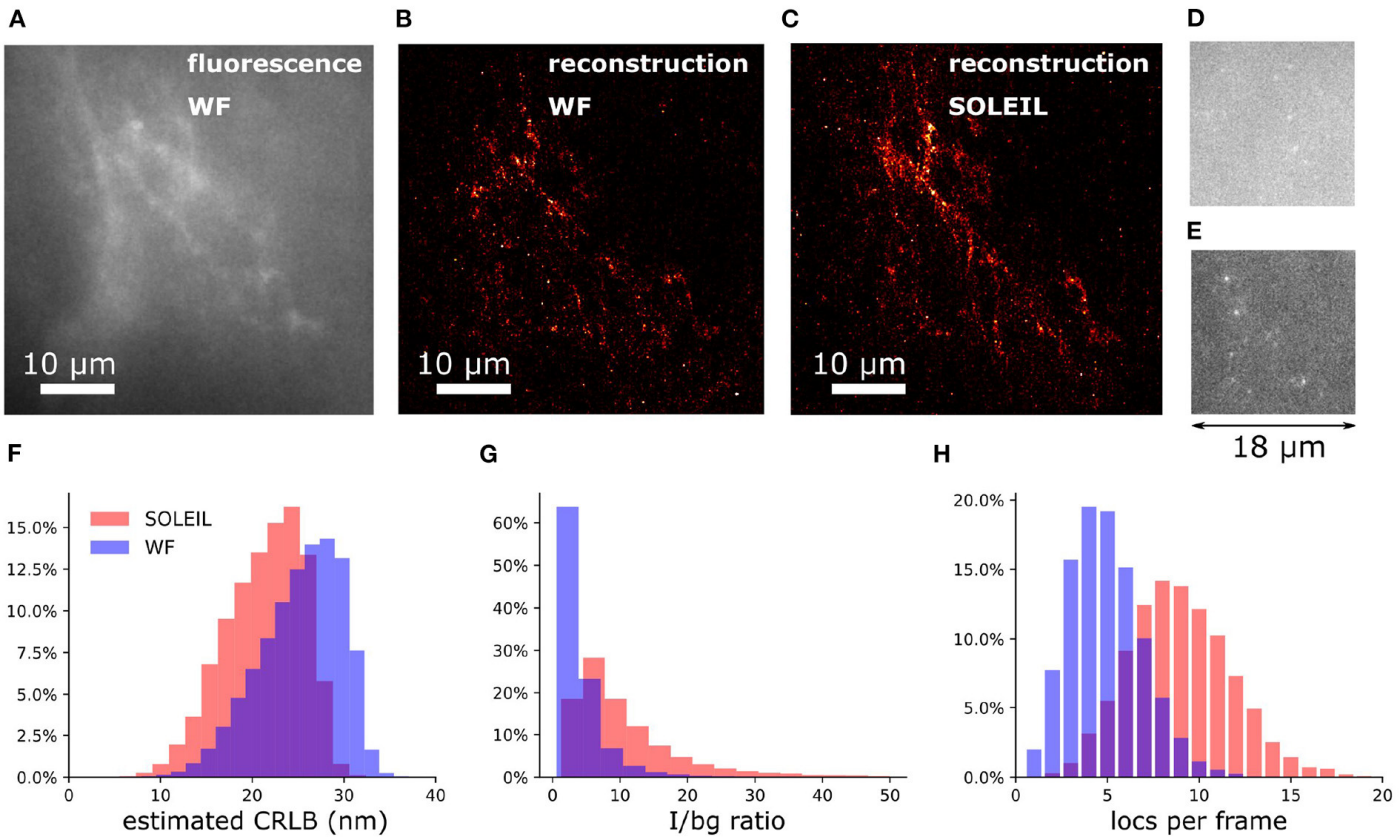
2D SMLM of a single neuron in an whole adult *Drosophila* brain. The imaging depth was 30 μm. **(A)** Fluorescence images of the same FOV of **(B,C)** with widefield microscopy. The fluorescence signal was acquired with low laser intensity to avoid blinking. **(B)** SMLM reconstruction of *Drosophila* brain with widefield microscopy. **(C)** SMLM reconstruction of *Drosophila* brain with SOLEIL microscopy. **(D,E)** Raw dSTORM images with widefield and SOLEIL microscopy. The images was plot in same contrast to fairly visualize the background reduction. **(F–H)** The estimated CRLB, I/bg ratio, and number of localization per frame (locs per frame) of widefield and SOLEIL microscopy.

To demonstrate our methodology in deep tissue, we imagined a single neuron in the brain of adult *Drosophila melanogaster*. We observed that the background in the brain tissue was significantly high, which demonstrates the importance of the optical sectioning delivered by SOLEIL (Figures 12D,E). The fluorescent signal from the expression of membrane-associated protein (myr-SNAP) under control of MB077c-Gal4 were acquired (Figure 12C) and corresponding SMLM reconstructions were made (Figures 12A,B). The quantitative comparison between SOLEIL and widefield microscopy revealed significant statistical improvements (Figures 12F–H). For SOLEIL microscopy, the median value of estimated CRLB was 21.8 nm and for WF microscopy, the median value of estimated CRLB was 25.8 nm. The median I/bg ratio from WF microscopy was 2.97 and the median I/bg ratio from SOLEIL microscopy was 8.21, which is a 276% improvement. The number of localizations per frame was increased by around 200% when SOLEIL microscopy was used for imaging.

### 4.6. 3D SMLM of a single neuron in adult *Drosophila* brains

**Figure 13:**
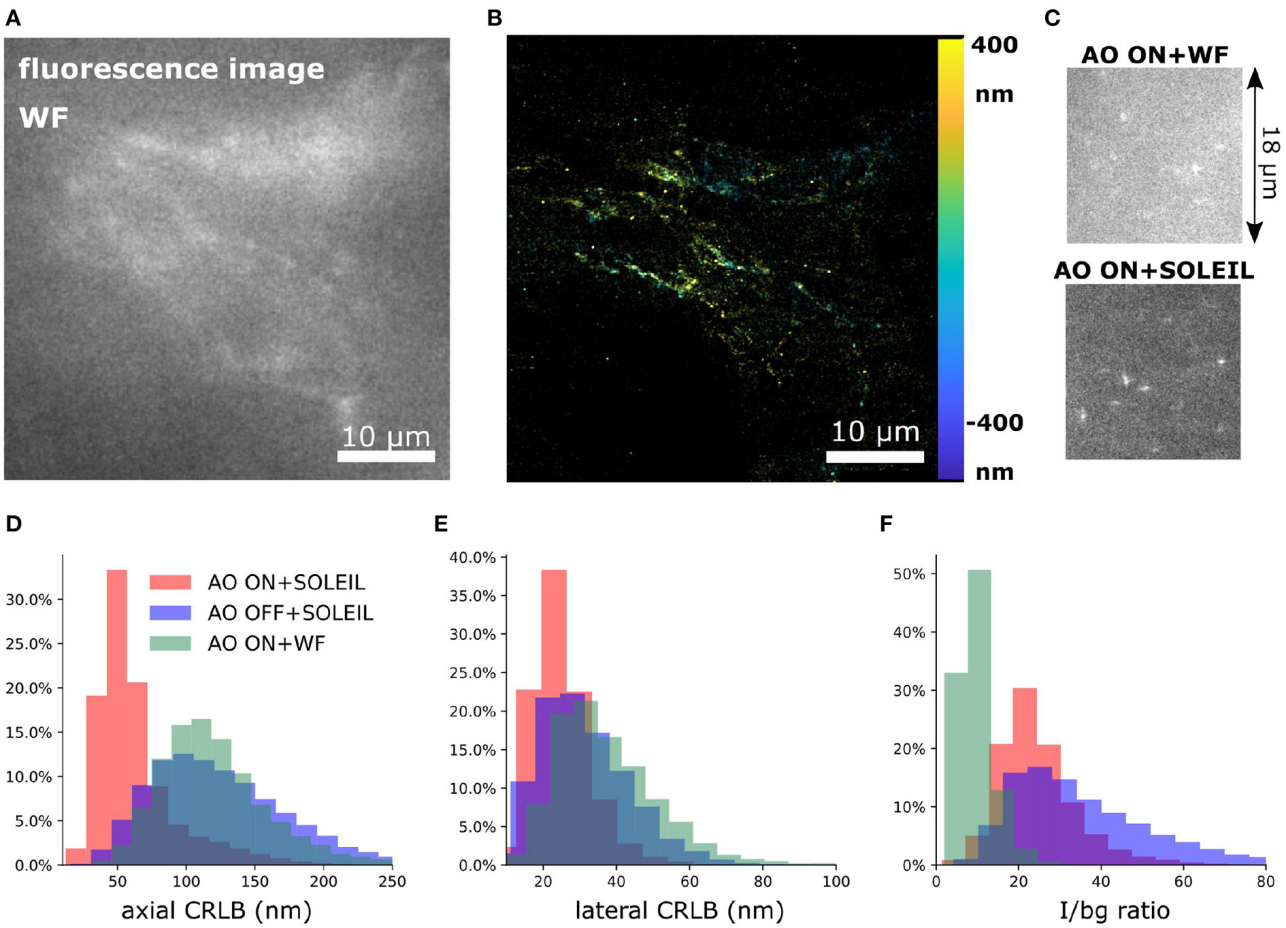
3D SMLM of a single neuron in a whole adult *Drosophila* brain. **(A)** The fluorescence signal of a part of the dendritic field of the single neuron with widefield microscopy. The data is acquired at a depth of 30 μm. **(B)** 3D SMLM reconstruction with SOLEIL and AO correction. **(C)** Raw camera image with widefield microscopy and SOLEIL microscopy. **(D–F)** Quantitative comparison of the axial CRLB, lateral CRLB, and I/bg ratio. The reconstruction images of AO OFF+SOLEIL and AO ON+WF are in (Figure 14).

**Figure 14:**
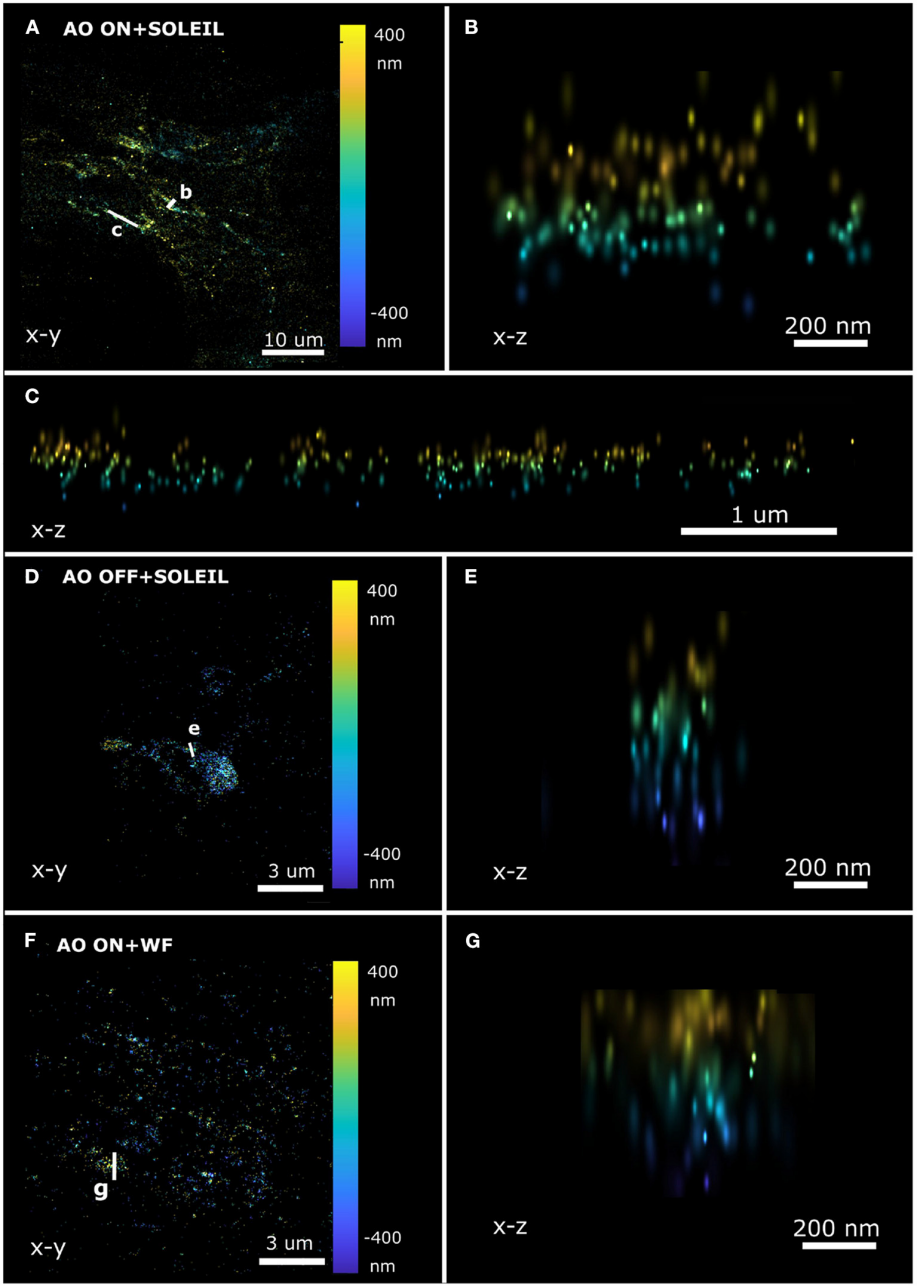
Axial SMLM reconstruction cross-section of a single neuron in a whole adult *Drosophila* brain. **(A–C)** SOLEIL illumination with AO correction. **(A)** Lateral view and axial cross-section **(B,C)**. **(D,E)** SOLEIL illumination without AO correction. **(D)** Lateral view and **(E)** axial cross-section. **(F,G)** Widefield illumination with AO correction. **(F)** Lateral view and **(G)** axial cross-section.

To perform 3D SMLM in adult *Drosophila* brains, we combined SOLEIL illumination, sensorless AO correction, and *in-situ* PSF calibration. SOLEIL microscopy resulted in a significant background reduction (Figures 13A,C). To demonstrate the importance of AO correction and SOLEIL illumination, three different situations were benchmarked: AO correction with SOLEIL microscopy (AO ON+SOLEIL), SOLEIL microscopy alone (AO OFF+SOLEIL), and AO correction with widefield microscopy (AO ON+WF) (Figures 13B, 14). The need for AO correction and the use of SOLEIL microscopy was evident from visual inspection of the corresponding reconstructions (Figures 13B, 14). The quantitative comparison between the three different situations also demonstrated several statistical improvements. The median axial CRLB value was improved by about 200 % (54.5, 115.7 and 114.1 nm; AO ON+SOLEIL, AO OFF+SOLEIL and AO ON+WF, respectively), suggesting that both AO correction and SOLEIL illumination are necessary for acquiring high-resolution 3D reconstruction image in *Drosophila* brains. The median lateral CRLB value was also slightly decreased (23.8, 29.3, and 34.7 nm; AO ON+SOLEIL, AO OFF+SOLEIL, and AO ON+WF, respectively). Lastly, the median value of the I/bg ratios were 22.95, 34.7, and 8.9 (AO ON+SOLEIL, AO OFF+SOLEIL, and AO ON+WF, respectively). These data suggest that AO-SOLEIL delivers an improvement for in tissue SMLM. In (Figure 14), we show the SMLM reconstruction with AO OFF+SOLEIL and AO OFF+WF. In the axial cross-section with AO ON+SOLEIL, we observed more fine structures while in the axial cross-sections with other two imaging conditions this is not visible.

## 5. Discussion and conclusion

In this work, to mitigate the sample-induced aberrations and high background effects when imaging thick samples, we synergetically combined sensorless adaptive optics (AO), *in-situ* 3D-PSF calibration, and a single-objective lens inclined light sheet microscope (SOLEIL) into a new methodology (AO-SOLEIL). We have demonstrated that SOLEIL can reject the out-of-focus fluorescence and thereby increase the I/bg ratio, localization precision, and number of detected spots per frame (Figures 1B–D). SOLEIL does not need a customized sample holder and only uses a single objective lens in the whole system thereby it is easier to cooperate adaptive optics element in the emission path. This feature makes the system accessible to non-expert users. We analyzed the benefit of aberration correction for 2D and 3D SMLM. In 2D SMLM, aberration correction can sharpen the PSF, which delivers better FRC resolution and more single-molecules can be detected (Figure 10). In 3D SMLM, we experimentally demonstrated that aberration correction can improve the axial CRLB (Figures 3, 13). A pitfall of using sensorless AO on three-dimensional structures is that the focal plane shifts during the correction of spherical aberration. This effect is predominant with the cross-talk between defocus and spherical aberration. To compensate for the induced defocus aberration and minimize the shift of the focal plane during the sensorless AO correction this cross-talk is calibrated (Figure 2).

We experimentally verified the improvement of our sensorless AO approach by imaging thick fluorescence bead sample (Figure 3). We demonstrated that sample-induced aberration can deteriorate the axial CRLB when imaging deep region of sample and aberration correction can restore the astigmatism PSF improving the axial CRLB. Furthermore, we demonstrated our approach is compatible with 2D and 3D SMLM dSTORM imaging. For 2D dSTORM, we found that the improvement in the FRC resolution is less significant in a thin sample (Figure 11) than in a thick sample (Figure 10). In the thin sample (17 μm deep; spheroid Caco2-BBE cells), we observed 11% of improvement in the FRC resolution (Figure 11C) and in the thick samples (65 μm deep; Caco2-BBE cells with artificial layer), we observed 47 % improvement in the FRC resolution (Figure 10H). For the 3D SMLM, we combined SOLEIL and sensorless AO method in *Drosophila* brain imaging to achieve the optimal axial CRLB. We performed the imaging with three different conditions (AO ON+SOLEIL, AO OFF+SOLEIL, and AO ON+WF) and benchmarked the estimated axial CRLB. We found that AO ON+SOLEIL achieves around 200% better estimated axial CRLB than in the other two situations, which suggests that both AO correction and SOLEIL illumination improves the SMLM when imaging tissue samples.

We anticipate that our approach can be used to image the whole adult *Drosophila* brain. We foresee that for imaging deeper in tissue the raw data quality can be improved by using photoactivation as an alternative to dSTORM, because photoactivation does not rely on a specialized buffer to penetrate the tissue (Betzig et al., 2006; Hess et al., 2006). Furthermore, photoactivation is compatible with clearing tissue methods, which will significantly reduce scattering of the illumination and emission light (Lin et al., 2019).

In our experiments, the imaging time was limited by the stability of the tissue. We hypothesize that the agarose gel gradually heats up during image acquisitions by the excitation laser, which causes thermal expansion and thus sample drifts. Therefore, the brain could be held in place by alternatives, such as by poly-L-lysine treated coverslips. Nevertheless, our synergistic approach enables super-resolution imaging with single color in sparsely-labeled neurons in adult *Drosophila* brains. To enable relevant 3D SMLM experiments, multi-color imaging would greatly enhance future studies addressing subcellular and molecular localizations of candidates of interest.

## Data availability statement

The raw data supporting the conclusions of this article will be made available by the authors, without undue reservation.

## Author contributions

All authors listed have made a substantial, direct, and intellectual contribution to the work and approved it for publication.

## Funding

S-TH and CS were supported by the Netherlands Organisation for Scientific Research (NWO), under NWO START-UP project no. 740.018.015 and NWO Veni project no. 16761. MS, DJ, and LK were supported by NWO, under FOM Neurophotonics project no. 16NEPH01. AL and LN were supported by the Swiss National Science Foundation SNSF Assistant Professor award (176855 and 211015), the International Foundation for Research in Paraplegia IRP (P180), and SNSF Spark (190919) to LN.

## Conflict of interest

The authors declare that the research was conducted in the absence of any commercial or financial relationships that could be construed as a potential conflict of interest.

## Publisher's note

All claims expressed in this article are solely those of the authors and do not necessarily represent those of their affiliated organizations, or those of the publisher, the editors and the reviewers. Any product that may be evaluated in this article, or claim that may be made by its manufacturer, is not guaranteed or endorsed by the publisher.
