# Supplementary material for: Adaptive optics in single objective inclined light sheet microscopy enables three-dimensional localization microscopy in adult Drosophila brains
Source: Front Neurosci. 2022 Oct 6;16:954949. doi: 10.3389/fnins.2022.954949 (PMC9583434; doi:10.3389/fnins.2022.954949)
Supplement: Supplementary file 1 [file Data_Sheet_1.pdf]

Supplementary Material

1 OPTICAL SYSTEM

The design of SOLEIL microscope is shown in (Hung et al., 2022). We used a stick-slip piezo stage as a sample stage (Smartact; x,y an SLC1730; z an SLC1720). The lightsheet was generated by an achromatic doublet cylindrical lens (Thorlabs, ACY254-250-B). Then, an achromatic doublet lens (Thorlabs, AC254-300-A-ML) formed a 4f-telescope with the cylindrical lens. To translate the lightsheet illumination, we placed a galvo mirror (Scanlab, dynAXIS 20 mm) at the pupil plane of the scan lens (TTL200MP, Thorlabs) to work as a scanning module. An achromatic doublet lens (Thorlabs, AC254-200-A-ML) and a reflective mirror (Thorlabs, BB1-E02) were placed on a translation stage (Thorlabs, XR25P/M), which allow us to adjust the angle of inclined lightsheet. A dichroic mirror (Semrock, Di03-R405/488/561/635-t1-25x36) separated the excitation and emission path. A 180 mm focal length tube lens (Olympus, SWTLU-C) was used to form a 60 times imaging system with objective lens (Olympus, UPlanSAPO 60x Oil NA 1.35). In the emission path, an achromatic lens with 200 mm focal length (AC254-200-A-ML) was assembled as a 4f-telescope with the tube lens, which conjugated the back focal plane of the objective lens to the plane of the deformable mirror (Alpao DM69-15). The deformable mirror allowed us to control the pupil phase in the emission path, which enables PSF engineering for 3D SMLM and sensorless AO correction. The deformable mirror was rotated by approximately $15°$ to reflect the emission light so that the emission light wouldn't reflect back to the original path. An emission filter (AHF, FF01-446/510/581/703-25) was used to filter out the back-reflected excitation laser. An achromatic doublet lens (AC254-200-A-ML) imaged the pupil plane at the plane of deformable mirror to the sCMOS camera (Andor Zyla 4.2). The sCMOS camera and galvo mirror were synchronized by using an Arduino micro-controller.

2 IN-SILICO SMLM DATA GENERATION

To test the stability and the performance of the sensorless AO algorithm, we built a SMLM data simulator (SDS) to simulate the blinking images in the presence of (Zernike) aberrations (Fig. S1). The pipeline of SDS consists of several steps. Firstly, we set up a 3D structure of the sample in the SDS (Fig. S1 (a)). In general, the structure can be any shape and in this experiment we set up the structure of the sample as tubulin-like. In the SDS simulator, the algorithm randomly chooses certain number of spots turning them to be the on-state and the others staying in the off-state. Then, SDS simulates the vector PSFs based on the input Zernike aberration, intensity and I/bg ratio (Fig. S1 (b)). The PSF simulation was done with ROI of 25 pixels$\times$ 25 pixels, which is 2.5 μm$\times$ 2.5 μm.

The high NA aberrated PSF was computed based on the pupil function (Siemons et al., 2018). The pupil function (Eq. S1) consists a phase aberration ($W\left(\vec{\rho}\right)$) and an aplantic amplitude factor ($A\left(\vec{\rho}\right)$) (Stallinga, 2015). The pupil function is described by a 91 pixels $\times$ 91 pixels array. The electric field (Eq. S2) is described as the Fourier transform of the pupil function ($v_{l,j}\left(\vec{\rho}\right)$) (Siemons et al., 2018). For numerical reasons we used the chirp z transform (Siemons et al., 2018; Bakx, 2002). We considered a free-rotating dipole and hence all polarization terms contribute to the PSF equally Eq. S3. Monochromatic light was used for simulating and the wavelength is 680 nm. The NA of objective lens we used is 1.35. The refractive index of immersion media is 1.52 and the refractive index of sample medium is 1.33. The pixel size of the simulated images is 108.33 nm. We neglect supercritical angle fluorescence (SAF) effects because they are neglectable away from the coverslip.

$$P\left(\vec{\rho}\right) = A\left(\vec{\rho}\right)\exp\left(i\frac{2\pi W\left(\vec{\rho}\right)}{\lambda}\right) \tag{S1}$$

,where $\vec{\rho}$ is the normalized pupil function radius, $P\left(\vec{\rho}\right)$ is the pupil function, $A\left(\vec{\rho}\right)$ is the aplantic amplitude factor, $W\left(\vec{\rho}\right)$ is the phase aberration, λ is the wavelength.

$$E_{l,j}\left(\vec{r}\right) = \int_{|\vec{\rho}|<1} d^2\rho P\left(\vec{\rho}\right) v_{l,j}\left(\vec{\rho}\right)\exp\left(-i\vec{k}\cdot\vec{r}\right) \tag{S2}$$

,where $E_{l,j}\left(\vec{r}\right)$ is the electric field with l, j as the direction of polarization (j emitter polarization projects to l polarization on pupil plane), $v_{l,j}\left(\vec{\rho}\right)$ is the vectorial factor (Stallinga, 2015), $\vec{k}$ is the wavevector.

$$PSF\left(\vec{r}\right) = \frac{1}{3}\sum_{l=x,y}\sum_{j=x,y,z}\left|E_{l,j}\left(\vec{r}\right)\right|^2 \tag{S3}$$

, where $PSF\left(\vec{r}\right)$ is the point spread function function.

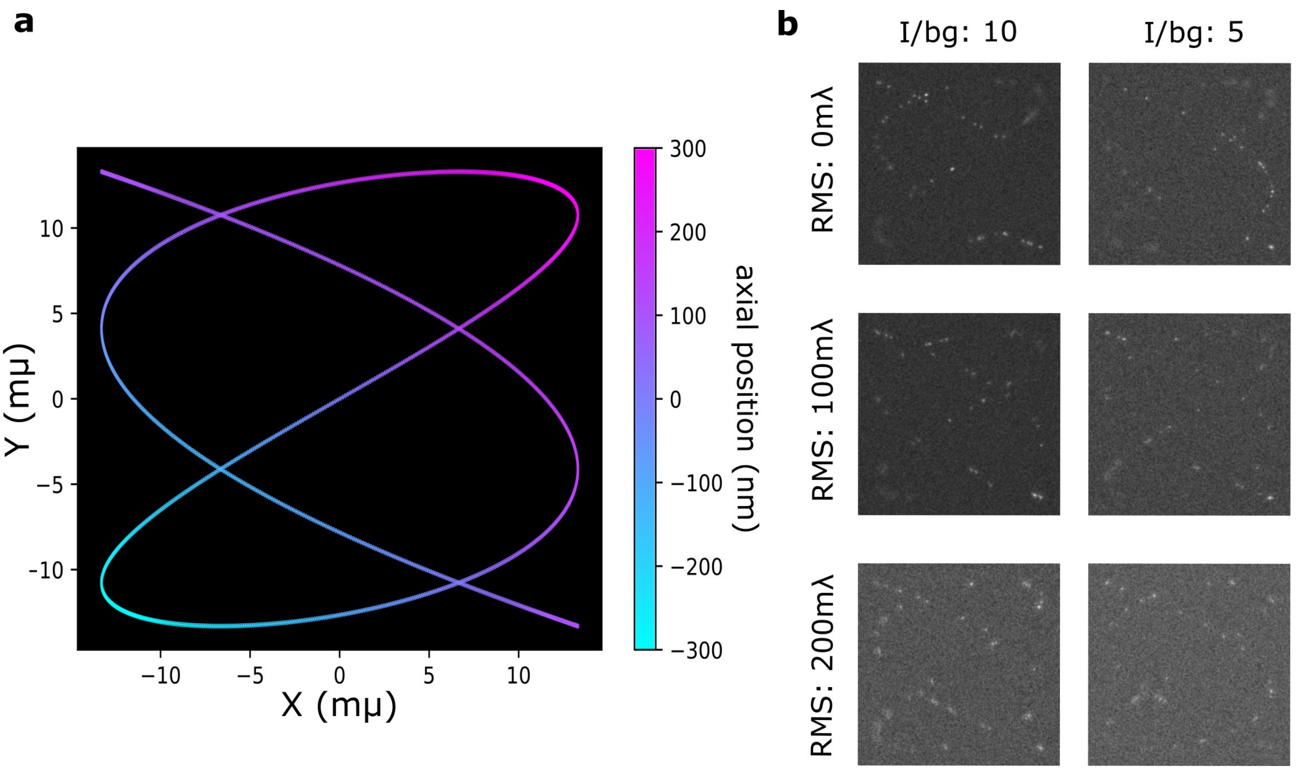

Figure S1. Blinking image simulator. (a) Tubulin structure for blinking image simulation. (b) Aberrated blinking image under different RMS value and I/bg ratio.

3 IMPACT OF AO CORRECTION ON THE LATERAL LOCALIZATION PRECISION FOR IN-SITU PSF ESTIMATION USING THE INSPR ALGORITHM

In this section the impact on the lateral CRLB is investigated. In Fig. 3, we show that AO correction can improve the axial CRLB. In Fig. S2 we found AO correction doesn't improve the lateral CRLB. In Fig. 8, it is also observed that AO correction has no significant improvement in lateral CRLB. Note that in Fig. 3,8 AO correction did improve the axial CRLB.

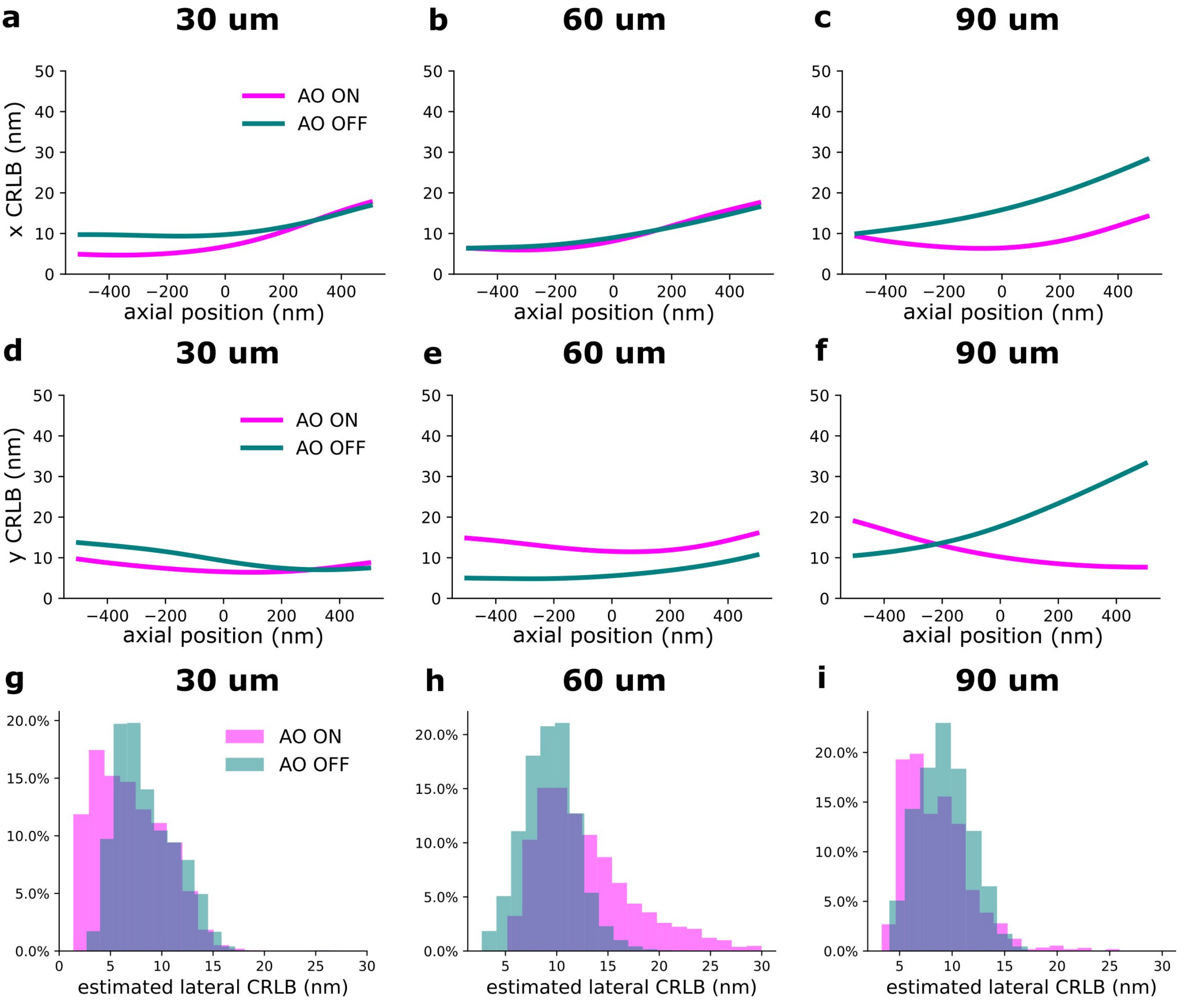

Figure S2. Lateral CRLB of the INSPR model before and after AO correction at different imaging depth. (a-f) The theoretical x,y CRLB based on the INSPR PSF model from Fig. 3 (a-c) with 3000 photon of intensity and 50 photons/pixel of background. (g-i) The distribution of estimated lateral CRLB at different imaging depths.

4 PREPARATION OF THE ARTIFICIALLY THICK CACO2-BBE SAMPLE

In this section, we show the flowchart on makig artificial thick Caco2-BBE sample. In Fig. S3 (a), we show the the schematic figure of the artificial thick sample from three views (top view and side view1,2). Fig. S3 (b) shows the material we used to make the artificially thick sample. Fig. S3 (c) shows detailed steps to create the artificial sample and how to add dSTORM buffer into the sample.

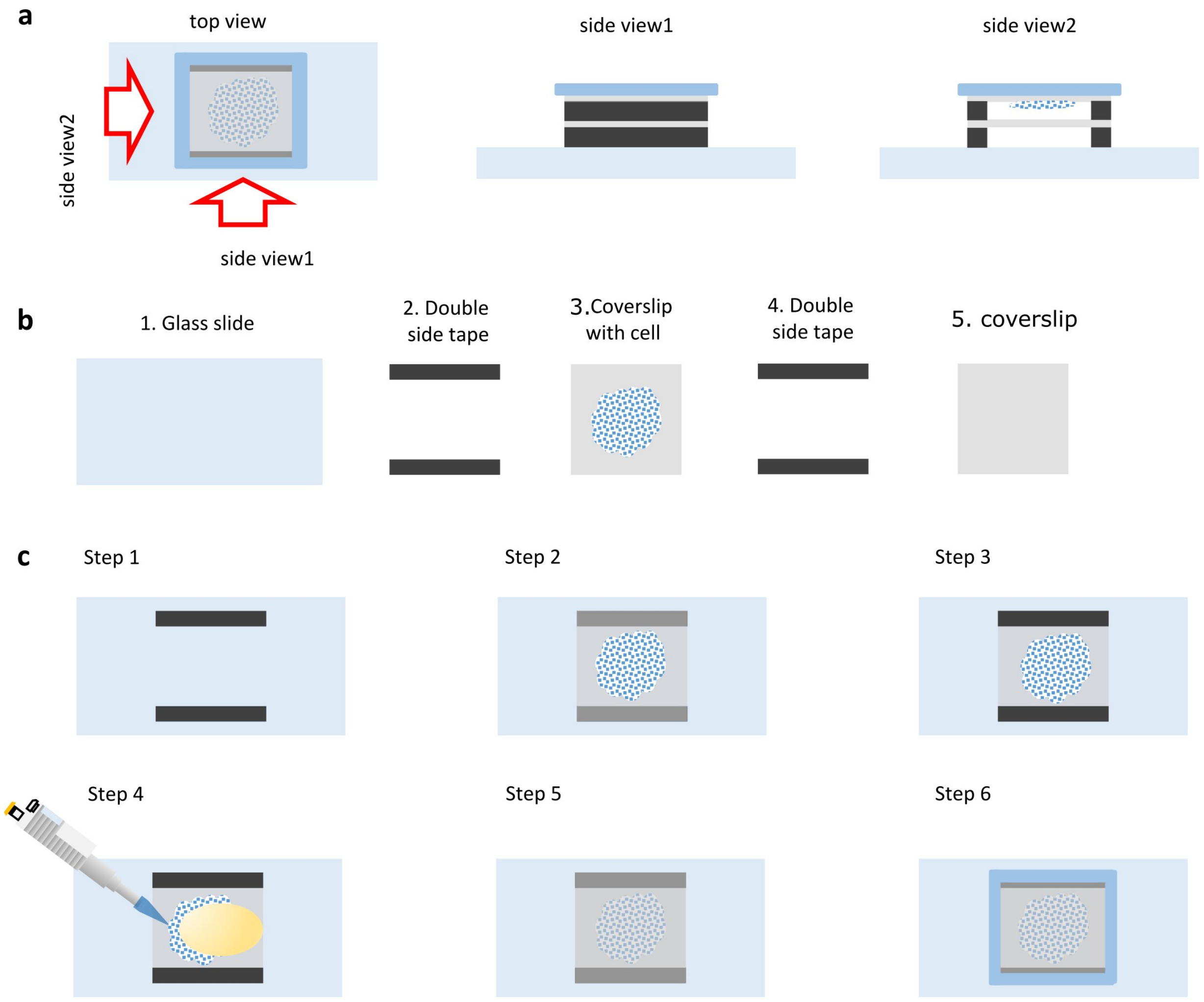

Figure S3. (a) The figure of thick STORM buffer layer Caco2-BBE sample and the sideview. (b) Material to make thick STORM buffer layer Caco2-BBE sample in (a,c). (c) Protocol to make thick STORM buffer layer Caco2-BBE sample. Step 1: put double side tape on glass slides, Step 2: attach a coverslip on the double side tape, Step 3: attach double side tape on the coverslip in Step 2, Step 4: add STORM buffer (yellow ellipse) in the middle of double side tape with pipette , Step 5: cover the cell with another coverslip, Step 6: seal the edge of coverslip by Twinsil (Picodent, Wipperfürth).

5 MEASURE THE THICKNESS OF DSTORM BUFFER LAYER

In the artificially thick Caco2-BBE sample, we added double sided tape as spacer between the cells and the coverslip to increase the thickness of the sample (Fig. S3), which can introduce additional spherical aberration. The thickness of the spacer was measured by the optical setup in Fig. S4 (a). A single mode coherent laser (640 nm) was launched from a reflected collimator and went through the 50:50 beam splitter focusing on the sample by an objective lens. The reflected light from the sample was acquired by the objective lens and went through the 50:50 beam splitter to the camera. When the incident light was focused on the interface between material, such as air-coverslip interface, the peak intensity of reflected light was much higher than the other situation. With this principle, we can identify the stage position from (b). The first interface the light can be focused on is the layer between air and the front side of the first coverslip, which is the P1 point in (b). The second interface can be focused is the back side of second coverslip, which is P2 point in (b). The third interface can be focused is the front side of second coverslip, which is the P3 point. The fourth interface can be focused is the back side of second coverslip. The distance between P2 and P3 is the distance of spacer we add in the sample, which is 64 μm.

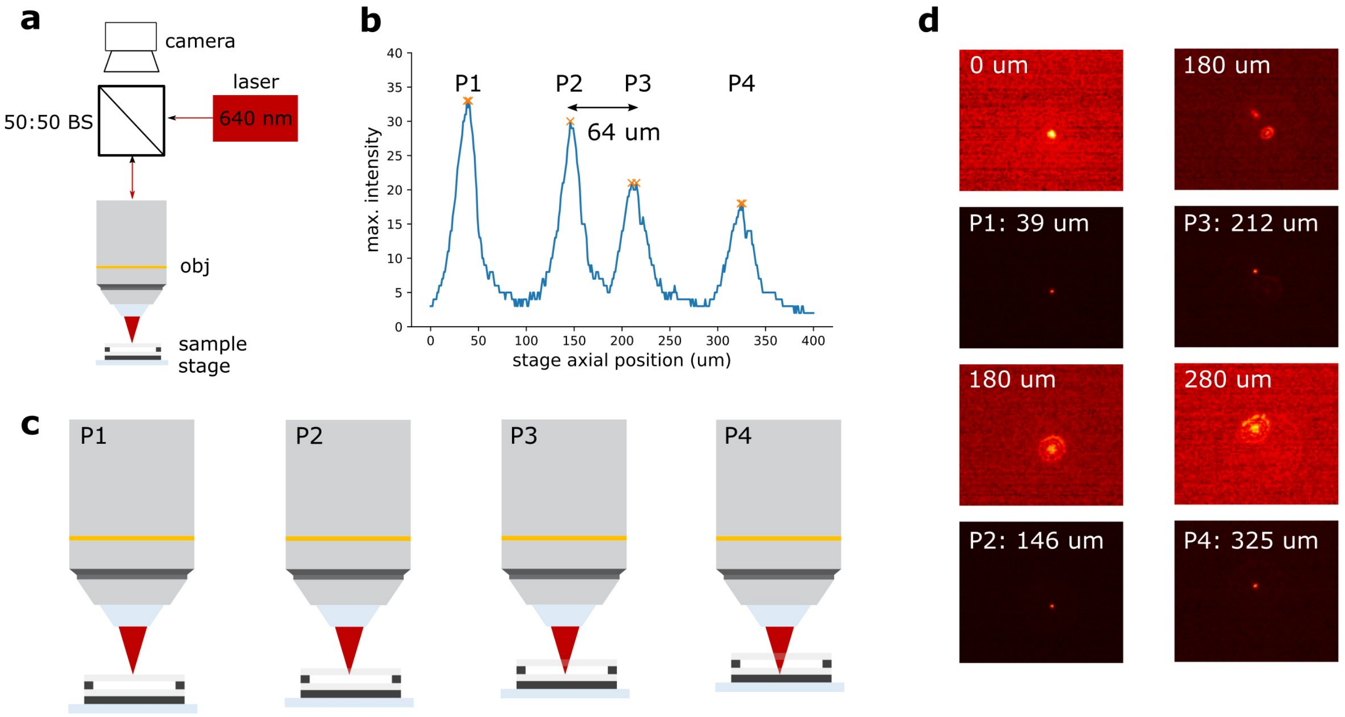

Figure S4. Measurement of the thickness of artificial Caco2-BBE sample. (a) optical setup for sample thickness measurement. 50:50 BS: 50:50 beam splitter (BS013, Thorlabs); camera: CMOS camera (IDS UI3070CP-M-GL); obj: 10X objective lens (UMPlanFL N 10X NA:0.3, Olympus); sample: measured sample; stage: piezo stage (Smactact, x,y an SLC1730; z an SLC1720); laser: 640 nm single mode laser (HLS635, Thorlabs). (b) the maximum image value at different stage position. (c) Schematic of stage position corresponding to the peak of (b). P1: the first peak in (b), where is light is focused at the interface of the front side of first coverslip and air. P2: the second peak in (b), where the light is focused at the interface of the back side of first coverslip. P3: the third peak in (b), where the light is focused at the front side of second coverslip. P4: the fourth peak in (b), where the light is focused at the back side of second coverslip. (d) the raw camera image at different stage position.

6 INFLUENCE OF SPHERICAL ABERRATION ON AXIAL CRLB

The spherical aberration can smooth the PSF along the axial dimension and this has an impact on the axial CRLB. To investigate the influence of spherical aberration on the axial CRLB, we simulated the the astigmatism and tetrapod PSF with additional spherical aberrations (Fig.S5 (a,c)) (Details of PSF simulation is in section 7). In the Fig.S5 (b,d), we computed the CRLB with a intensity of 1000 photons and a background of 50 photons/pixel and found that the spherical aberration can significantly decrease the CRLB of engineered PSF.

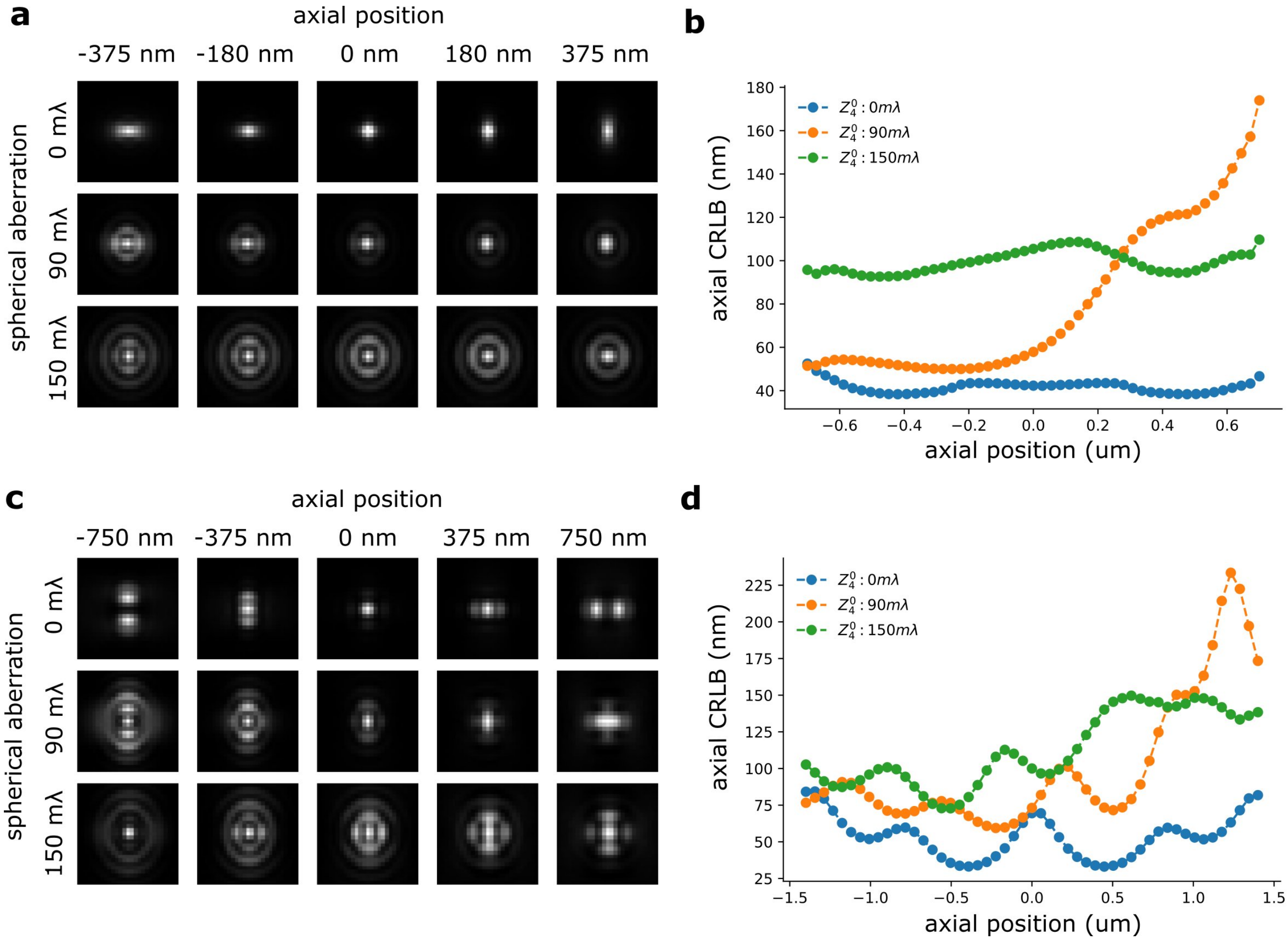

Figure S5. Influence of spherical aberration on axial CRLB. (a) Simulated astigmatism PSF (100 mλZ_2^2) with the addition of different level of spherical aberration. (b) The axial theoretical CRLB of the astigmatism PSF with addition of different level of spherical aberration. The intensity used for simulation is 1000 photons and the background is 50 photons/pixel. (c) Simulated tetrapod PSF (100 mλZ_2^4) with the addition of different level of spherical aberration. (b) The axial theoretical CRLB of the tetrapod PSF with addition of different level of spherical aberration. The intensity used for simulation is 1000 photons and the background is 50 photons/pixel.

7 INFLUENCE OF SPHERICAL ABERRATION ON THE 2D LOCALIZATION PRECISION

To understand the influence of spherical aberration on the 2D localization of single-molecules, we simulated PSFs with different amounts of spherical aberration (details is in section 7) and performed the localization by using Gaussian PSF model. We observed that the spherical aberration deteriorates the achievable localization (Fig. S6 (a)). The reported localization precision is the standard deviation of estimated position from 100 times of localization and we repeated this for 10 time for the errorbar in Fig. S6 (a). The spherical aberration can distort the PSF and the shape of the aberrated PSF doesn't match with a Gaussian function. The PSFs were simulated with the same intensity and background. However, the estimated intensity and background are biased in the presence of spherical aberration (Fig. S6 (c,d)). In the meanwhile, the I/bg decreases (Fig. S6 (e)). The reported CRLB and the estimation precision deteriorate by 40% as the spherical aberration (Z_4^0) increases to 120 mλ.

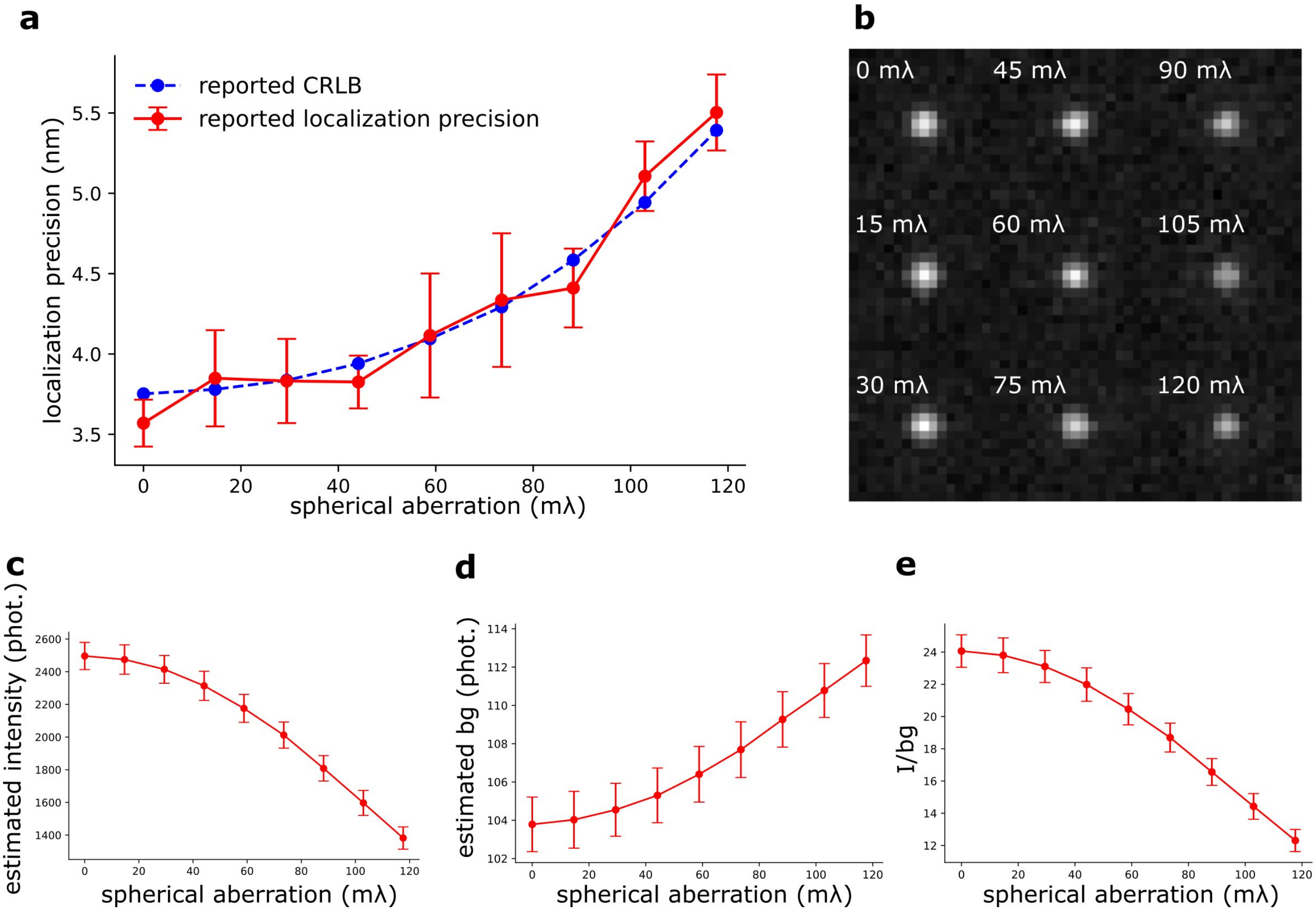

Figure S6. Influence of spherical aberration on the 2D localization precision. (a) the CRLB, reported localization precision versus amplitude of spherical aberration. (b) the simulated PSF with different amplitude of spherical aberration. (c-e) the influence of spherical on estimated intensity (c), background (d), and SBR (e).

8 CAMERA SETTING

In this research, we used Andor Zyla 4.2 SCMOS camera for imaging. For the dSTORM imaging, we used 501 pixels$\times$501 pixels, which corresponds to 54 μm$\times$54 μm FOV. The camera trigger was set to external trigger, which allows for external signal control from Arduino Micro-controller. In SOLEIL microscopy, the virtual confocal slit readout was achieved with a rolling shutter by activating the Andor SOLIS LightScan PLUS function in down sequential mode. The readout image is 16 bit format.

For initial aberration correction, the camera trigger mode was set to internal trigger and the camera was synchronized with DM through the customized Python script. The FOV was cropped to single PSF spot. The camera exposure time was 30 ms, which is 25 frames/second. The image readout was set to 16 bit mode.

For sensorless AO correction, the camera trigger mode was set to internal trigger and used customized Python script for controlling camera and DM. The camera readout is 16 bit. The FOV was cropped depend on the size of sample. In general, we ensured the FOV is larger than 30 μm$\times$30 μm.

REFERENCES

Bakx, J. L. (2002). Efficient computation of optical disk readout by use of the chirp z transform. *Appl. Opt.* 41, 4897–4903. doi:10.1364/AO.41.004897

Hung, S.-T., Cnossen, J., Fan, D., Siemons, M., Jurriens, D., Grußmayer, K., et al. (2022). Soleil: single-objective lens inclined light sheet localization microscopy. *Biomedical Optics Express* 13, 3275–3294. doi:10.1364/BOE.451634

Siemons, M., Hulleman, C. N., Thorsen, R. O., Smith, C. S., and Stallinga, S. (2018). High precision wavefront control in point spread function engineering for single emitter localization. *Optics Express* 26, 8397–8416. doi:10.1364/OE.26.008397

Stallinga, S. (2015). Effect of rotational diffusion in an orientational potential well on the point spread function of electric dipole emitters. *J. Opt. Soc. Am. A* 32, 213–223. doi:10.1364/JOSAA.32.000213
